# Assessing the reliability of hippocampal single-voxel proton magnetic resonance spectroscopy through intra-class effect decomposition

**DOI:** 10.1162/IMAG.a.1401

**Published:** 2026-10-16

**Authors:** Martin Nilsson, Jonathan Berrebi, Sahar Nikkhou-Aski, Bárbara Avelar-Pereira, Per Thorsell, Vlad Legendi, Marc Guitart-Masip, Goran Papenberg

**Affiliations:** Aging Research Center, Department of Neurobiology, Care Sciences, and Society, Karolinska Institutet and Stockholm University, Sweden; Stockholm University Brain Imaging Centre, Stockholm University, Stockholm, Sweden; Department of Nuclear medicine and Medical Physics, Karolinska University Hospital Solna, Stockholm, Sweden; Department of Clinical Science, Intervention and Technology, Karolinska Institutet, Stockholm, Sweden; Department of Psychiatry and Behavioral Sciences, School of Medicine, Stanford University, Stanford, CA, United States; Department of Cognitive Neuroscience and Philosophy, School of Bioscience, University of Skövde, Skövde, Sweden; Centre for Psychiatry Research, Department of Clinical Neuroscience, Karolinska Institutet, Stockholm, Sweden

**Keywords:** hippocampus, intra-class effect decomposition, reliability, magnetic resonance spectroscopy, semi-LASER

## Abstract

The hippocampus is crucial for learning, memory, and stress regulation, but is vulnerable to influences that compromise its structural and metabolic integrity. Proton magnetic resonance spectroscopy (MRS) enables in vivo quantification of hippocampal neurochemistry, yet the absolute and relative reliability of these measures has rarely been systematically evaluated. We assessed hippocampal metabolite estimates acquired with single-voxel MRS using a semi-adiabatic localization by adiabatic selective refocusing (semi-LASER) sequence at 3 T. Nineteen healthy adults (8 male; mean age = 40.9 ± 16.9 years) underwent four measurements across 2 consecutive days, with one session including an extended acquisition. Absolute reliability was quantified using within-subject coefficients of variation (CVs), and relative reliability was estimated with the Intra-Class Effect Decomposition (ICED) framework, which partitions variance into true-score, day-specific, session-specific, and residual components. Despite good spectral quality and high absolute reliability for several metabolites, relative reliability ranged from poor to good. Good relative reliability was observed only for water-referenced myo-inositol, total choline, and total creatine; no creatine-referenced metabolite reached this threshold. Increasing the number of transients increased signal-to-noise ratio but did not improve relative reliability; only creatine-referenced total N-acetylaspartate showed significant session-specific variance. Thus, high absolute reliability did not necessarily imply high relative reliability. Hippocampal MRS at 3 T may, therefore, be suitable for group-level or mean-level metabolite estimates, but appears less suitable for correlational, biomarker, or other individual-difference applications that require stable between-person differences. Methodological refinement, including improved voxel placement, motion correction, shimming, and quantification pipelines, may improve the relative reliability of hippocampal MRS.

## 1 Introduction

The hippocampus is a critical brain region for learning, memory, and stress regulation, but its high plasticity and metabolic demand, while essential for adaptive function, also render it particularly vulnerable to biological and environmental influences (Bartsch & Wulff, 2015; Mcewen, 1999; Small et al., 2011). Multiple interacting mechanisms, including synaptic and dendritic remodeling (Bartsch & Wulff, 2015; Burke & Barnes, 2006), neuroinflammation (Barrientos et al., 2015), and metabolic dysregulation (Fan et al., 2017), can compromise hippocampal integrity and function. Consequently, hippocampal alterations are common across several neurological and psychiatric conditions, including Alzheimer’s disease, major depressive disorder, post-traumatic stress disorder, and temporal lobe epilepsy (Belleau et al., 2019; Braak & Braak, 1991; Moodley & Chan, 2014). These vulnerabilities highlight the need for in vivo methods capable of quantifying underlying neurochemical and metabolic processes.

Proton magnetic resonance spectroscopy (MRS) provides a unique, noninvasive tool to quantify neurochemical composition in vivo (Öz et al., 2014). In the hippocampus, MRS can measure a wide range of metabolites, such as N-acetylaspartate (NAA), creatine, choline, myo-inositol (mI), and glutamate, which serve as markers of neuronal integrity, energy metabolism, membrane turnover, gliosis and inflammation, and glutamatergic metabolism (Chang et al., 2013; Cleeland et al., 2019; Duarte et al., 2012; Rae, 2014; Ross & Bluml, 2001; Song et al., 2021). These metabolites have been studied in a range of neurological and psychiatric disorders, including Alzheimer’s disease, epilepsy, schizophrenia, and major depression (de Diego-Adeliño et al., 2013; Husarova et al., 2012; Hutcheson et al., 2012; Kuzniecky et al., 2001; H. Liu et al., 2021). Yet the hippocampus remains a technically challenging target, where its small size causes partial volume effects and its proximity to air–tissue interfaces produces artifacts such as poor shimming and signal loss (Choi & Frahm, 1999). These limitations highlight the need for rigorous acquisition and analysis methods (Wilson et al., 2019) to ensure reliable hippocampal metabolite quantification (Bednařík et al., 2015).

Reliable hippocampal metabolite estimates are particularly important for detecting meaningful neurochemical signatures and for establishing robust biomarkers in longitudinal and individual-differences research. The most commonly reported reliability index in hippocampal MRS studies is the coefficient of variation (CV), which indexes absolute reliability, defined as the precision of repeated measurements (Atkinson & Nevill, 1998). CV is calculated as the within-subject standard deviation relative to the mean (Kreis et al., 2021). Prior reproducibility studies have demonstrated low CVs, indicating high absolute reliability, for three to seven hippocampal metabolites under optimized acquisition protocols (Allaïli et al., 2015; Bednařík et al., 2015; Gajdošík et al., 2021; Zhang et al., 2018). However, CV does not reveal whether individuals maintain stable rank ordering across repeated measurements. This property, known as relative reliability, is instead captured by the intraclass correlation coefficient (ICC), which represents the proportion of total variance attributable to between-person differences rather than measurement error (Atkinson & Nevill, 1998; Shrout & Fleiss, 1979).

ICC complements CV (Brandmaier et al., 2018) and is critical for longitudinal investigation and when linking metabolite levels to behavioral or clinical phenotypes, as meaningful associations require stable between-person variability (Atkinson & Nevill, 1998; Gell et al., 2024). This is particularly relevant given the growing emphasis in neuroscience and psychiatry on dimensional approaches aimed at capturing individual variability in brain–behavior relationships (Cuthbert & Insel, 2013; Insel et al., 2010; Marek et al., 2022). However, evidence on ICC in hippocampal MRS is scarce. To our knowledge, Allaïli et al. (2015) is the only prior hippocampal MRS study to report ICCs, finding low values with standard acquisition lengths and ICCs of 0.38-0.75 only with extended acquisitions (768 transients). This suggests that increasing acquisition length may improve relative reliability, consistent with the expected SNR gain from additional transients (Allaïli et al., 2015). However, very long acquisitions may not be feasible for patients or certain populations (e.g., older adults) because longer scan times may increase scanner burden and vulnerability to motion. Moreover, Allaïli et al. used localization by adiabatic selective refocusing (LASER), whereas the semi-adiabatic variant, semi-LASER, is recommended in consensus guidance for clinical proton MRS (Wilson et al., 2019) and has been shown to produce better absolute reliability than standard sequences such as PRESS (Deelchand et al., 2018). It, therefore, remains unclear whether the reliability benefit of extended acquisition length observed with LASER generalizes to semi-LASER.

To address this gap, we applied the Intra-Class Effect Decomposition (ICED) framework, which extends the conventional ICC by partitioning variance into stable between-person differences, session-specific effects, day-specific effects, and residual error (Brandmaier et al., 2018). This allowed us to assess relative reliability and characterize sources of measurement error under a semi-LASER protocol, with a specific focus on whether increasing acquisition length improves relative reliability.

## 2 Methods

### 2.1 Participants

Nineteen healthy volunteers (8 males; mean age = 40.9 ± 16.9 years) participated. They had normal or corrected-to-normal vision and hearing, no history of neurological disorders, and no contraindications for MRI (e.g., implants, claustrophobia). All participants provided written informed consent. The Swedish Ethical Review Authority approved the study, which was conducted in accordance with the Declaration of Helsinki.

### 2.2 Procedure

After providing written informed consent, participants were placed in the scanner in a supine position with the head slightly tilted to optimize hippocampal visibility. Foam padding was used to stabilize the head and minimize motion. Participants were instructed to remain still, minimize swallowing during acquisitions, and stay awake throughout the session. On Day 1, they completed two scans with 128 transients (D1M1 and D1M2). On Day 2, they completed two additional scans, one with 256 transients (D2M1) and one with 128 transients (D2M2), with order counterbalanced across participants. The 256-transient acquisition was included to introduce a structured acquisition difference for the ICED variance-decomposition model. For each scan, shimming and voxel placement were repeated independently.

### 2.3 Data acquisition

All scans were acquired on a Siemens MAGNETOM Prisma 3T scanner (software version XA60) using a 20-channel head coil at the Stockholm University Brain Imaging Center. High-resolution sagittal magnetization-prepared rapid gradient echo (MPRAGE) images were collected (voxel size = 1 mm³, TR = 2300 ms, TE = 2.98 ms, TI = 900 ms, flip angle = 9°, field of view = 256 × 256 mm², GRAPPA factor = 2; acquisition time = 5:21 min).

Proton MRS was acquired with a semi-LASER sequence from the CMRR Spectroscopy Package (Deelchand et al., 2021, 2022; Öz & Tkáč, 2011) (TR = 5000 ms, TE = 30 ms [TE1 = 8 ms, TE2 = 12 ms, and TE3 = 10 ms]). The voxel of interest measured 27 × 12 × 13 mm³ and was manually placed in the right hippocampus on each participant’s T1-weighted anatomical image. Positioning was guided by anatomical landmarks to align the voxel along the longitudinal axis and to cover the hippocampal body while avoiding inclusion of cerebrospinal fluid (CSF) as far as possible. Voxel placement was performed by a single trained operator to ensure consistency across participants and sessions. Voxel-placement consistency across measurement occasions was assessed using Dice coefficients (see Section 2.8.3 and Supplementary Table S13). Due to known susceptibility differences between tissues in the anterior hippocampus (Arslanoglu et al., 2004), the most anterior portions of the hippocampus and the amygdala were not included in the voxel. An example placement is shown in Figure 1. Excitation and refocusing pulses were 2000 µs and 4500 µs (flip angles 90° and 180°). Outer-volume suppression was combined with Variable Power and Optimized Relaxation Delays (VAPOR) water suppression consisting of seven frequency-selective pulses with variable flip angles (maximum ≈ 76°, bandwidth ≈70 Hz). Spectra were acquired with a 6000 Hz bandwidth and 2048 points, using either 128 averages (10:40 min) or 256 averages (21:20 min), depending on the session. Four unsuppressed water scans were collected for eddy-current correction and metabolite quantification. Prior to acquisition, B0 shimming was performed with FAST(EST)MAP (Gruetter & Tkáč, 2000).

**Fig. 1.**
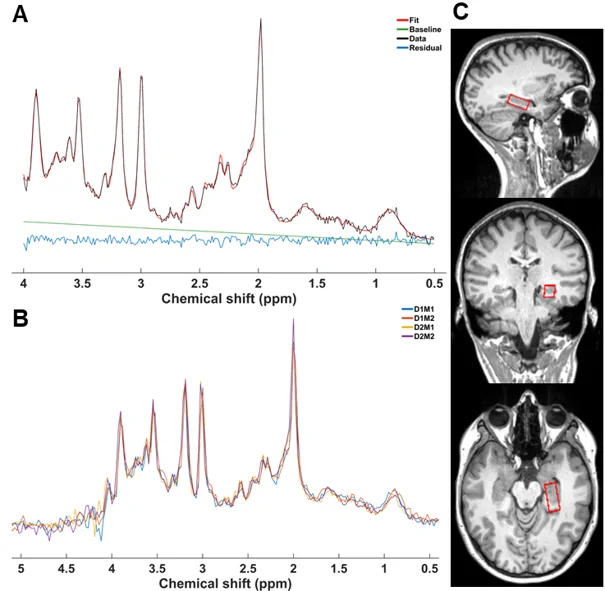
Voxel placement and representative spectra from repeated measurements. Representative spectra and voxel placement from one participant. (A) Example fitted spectrum from one measurement, showing the LCModel fit, baseline, measured data, and residual. (B) Processed spectra from four repeated measurements in the same participant (58-year-old female). No additional scaling or smoothing was applied for visualization. (C) Volume of interest positioned in the right hippocampus, shown in sagittal, coronal, and axial views.

### 2.4 Data processing

MRS data were processed with the Osprey toolbox v2.9.2 (Oeltzschner et al., 2020), which is implemented in MATLAB (MATLAB R2022a, MathWorks, Natick, MA, USA) and performs single-shot frequency and phase correction, eddy-current correction, elimination of residual water, baseline correction, and tissue segmentation. Spectral fitting was conducted in LCModel (linear-combination model; v6.3-1L) (S. W. Provencher, 1993) over the 0.2–4 ppm range using a simulated semi-LASER basis set provided by CMRR (University of Minnesota). The basis set included alanine (Ala), ascorbate (Asc), aspartate (Asp), creatine (Cr), γ-aminobutyric acid (GABA), glucose (Glc), glutamine (Gln), glutamate (Glu), glycine (Gly), glycerophosphocholine (GPC), glutathione (GSH), myo-inositol (mI), scyllo-inositol (sI), lactate (Lac), phosphocreatine (PCr), phosphocholine (PCho), phosphoethanolamine (PE), N-acetylaspartate (NAA), N-acetylaspartylglutamate (NAAG), and taurine (Tau). Macromolecule and lipid contributions were modeled using Osprey’s default parameterization. Summary metabolites, including total creatine (tCr = Cr + PCr), total NAA (tNAA = NAA + NAAG), total choline (tCho = GPC + PCho), Glx (Glu + Gln), and Glc+Tau, were automatically calculated by Osprey. Metabolite concentrations were quantified as both tCr-referenced and water-referenced values. The water-referenced estimates were tissue- and relaxation-corrected according to the Gasparovic method (Gasparovic et al., 2006), using gray matter (GM), white matter (WM), and CSF fractions derived from SPM12 (Ashburner & Friston, 2005) segmentation, yielding tissue-corrected, water-scaled values expressed in mmol/kg tissue water (approximated concentration units).

### 2.5 Data quality and metabolite selection

All spectra met minimum quality criteria (Wilson et al., 2019): signal-to-noise ratio (SNR > 10, calculated in LCModel relative to the NAA peak) and linewidth (LW < 12 Hz, calculated in Osprey as the full width at half maximum (FWHM) of a Lorentzian peak model for the water peak between 4.4 and 5.0 ppm). Visual inspection further identified artifacts from poor water suppression and frequency instability, resulting in the exclusion of five out of 76 spectra. Each spectrum reflected the average of 128 or 256 transients from a given session, and artifact evaluation was conducted on these averaged spectra.

Metabolites with mean CRLB values ≤ 30% across all measurements were included (Bednařík et al., 2020). CRLB values were used as conventional fit-quality indices and as lower bounds on model-based uncertainty in the fitted metabolite amplitudes, and should therefore be interpreted cautiously, particularly for water-scaled estimates that also depend on tissue correction and relaxation assumptions (Instrella & Juchem, 2024; Landheer & Juchem, 2021). Metabolites showing strong negative correlation coefficients (r < −0.5) were reported as combined sums (Eftekhari et al., 2025; S. Provencher, n.d.): Cr and PCr (r = −0.924) as tCr, NAA and NAAG (r = −0.677) as tNAA, and Glc and Tau (r = −0.648) as Glc+Tau. Gln and Glu showed a weak correlation (r = −0.228) and were retained separately. Glx was additionally included because it is a commonly reported composite measure, but should not be interpreted as independent from Glu and Gln. The selected metabolites are shown in Figure 6 and Supplementary Table S9.

### 2.6 Absolute reliability

Absolute reliability was assessed using the CV, calculated for each metabolite as the within-subject standard deviation across the four measurements divided by the corresponding mean. For participant i, this was defined as

$$CV_{i}=\frac{\text{SD}\left(\text{D}1\;\text{M}1_{i},\text{D}1\;\text{M}2_{i},\text{D}2\;\text{M}1_{i},\text{D}2\;\text{M}2_{i}\right)}{\text{mean}\left(\text{D}1\;\text{M}1_{i},\text{D}1\;\text{M}2_{i},\text{D}2\;\text{M}1_{i},\text{D}2\;\text{M}2_{i}\right)}.$$

### 2.7 Relative reliability

ICED was used to partition the observed between-person variance into orthogonal components reflecting distinct sources of measurement variability. Outliers were screened separately for each metabolite using univariate (±3 SD) criteria and multivariate Mahalanobis-distance criteria. For the Mahalanobis-distance screening, observations were classified as outliers if they exceeded the cutoff expected for four repeated measurements at p < .001. No observations met either criterion.

Structural equation models were fitted in R version 4.5.0 (R Core Team, 2025). using lavaan (Rosseel, 2012), following the ICED framework (Brandmaier et al., 2018) and based on scripts provided by Wenger et al. (2022). For participant i, the four repeated measurements were specified as

$$D1\;M1_{i}\;=T_{i}\;+D1_{i}\;+S12_{i}\;+E1_{i},$$

$$D1\;M2_{i}\;=T_{i}+D1_{i}\;+S12_{i}\;+E2_{i},$$

$$D2\;M1_{i}\;=T_{i}\;+D2_{i}\;+S3_{i}\;+E3_{i},$$

$$D2\;M2_{i}\;=T_{i}+D2_{i}\;+S4_{i}\;+E4_{i}.$$

Here, *$D1\;M1_{i}$*, *$D1\;M2_{i}$*, *$D2\;M1_{i}$*, and $D2\;M2_{i}$ denote the observed measurements for participant i. $T_{i}$ denotes the latent true-score factor, $D1_{i}$ and $D2_{i}$ denote latent day factors, $S12_{i}$, $S3_{i}$, and $S4_{i}$ denote latent session factors, and $E1_{i}$ to $E4_{i}$ denote latent residual factors. The latent factors were specified as mutually orthogonal, and their variances were parameterized as

$$\text{Var}\left(T_{i}\right)=\sigma_{T}^{2},$$

$$\text{Var}\left(D1_{i}\right)=\text{Var}\left(D2_{i}\right)=\sigma_{D}^{2},$$

$$\text{Var}\left(S12_{i}\right)=\text{Var}\left(S3_{i}\right)=\text{Var}\left(S4_{i}\right)=\sigma_{S}^{2},$$

$$\text{Var}\left(E1_{i}\right)=\text{Var}\left(E2_{i}\right)=\text{Var}\left(E3_{i}\right)=\text{Var}\left(E4_{i}\right)=\sigma_{E}^{2},$$

with variance parameters constrained to non-negative values (≥ 0.0001). This parameterization defines a single variance parameter for each source of variation and yields a decomposition of total observed variance into $\sigma_{T}^{2}\;+\sigma_{D}^{2}\;+\sigma_{S}^{2}\;+\sigma_{E}^{2}$, allowing each component to be interpreted directly as a contribution to total variance (Brandmaier et al., 2018).

The ICC was defined as the proportion of total variance attributable to between-person differences. In the present model, this corresponds to the ratio of true-score variance to total observed variance, that is,

$$ICC=\frac{\sigma_{T}^{2}}{\sigma_{T}^{2}\;+\sigma_{D}^{2}\;+\sigma_{S}^{2}\;+\sigma_{E}^{2}}.$$

An ICC close to 1 indicates high relative reliability, meaning that a large proportion of total variance is attributable to between-person differences. For descriptive interpretation, values < 0.40 are considered poor, 0.40–0.59 fair, 0.60–0.74 good, and ≥ 0.75 excellent (Baeshen et al., 2020; Cicchetti, 1994).

Variance components were estimated using full-information maximum likelihood. Nested models were specified by fixing $\sigma_{T}^{2}$, $\sigma_{D}^{2}$, or $\sigma_{S}^{2}$ to zero and compared with the full model using likelihood-ratio tests to assess whether each variance component differed from zero. The residual variance component $\sigma_{E}^{2}$ was evaluated using a Wald test (Brandmaier et al., 2018). For interpretability, the estimated variance components were normalized by their sum:

$$VarT=\frac{\sigma_{T}^{2}}{\sigma_{T}^{2}\;+\sigma_{D}^{2}\;+\sigma_{S}^{2}\;+\sigma_{E}^{2}},$$

$$VarD=\frac{\sigma_{D}^{2}}{\sigma_{T}^{2}\;+\sigma_{D}^{2}\;+\sigma_{S}^{2}\;+\sigma_{E}^{2}},$$

$$VarS=\frac{\sigma_{S}^{2}}{\sigma_{T}^{2}\;+\sigma_{D}^{2}\;+\sigma_{S}^{2}\;+\sigma_{E}^{2}},$$

$$VarE=\frac{\sigma_{E}^{2}}{\sigma_{T}^{2}\;+\sigma_{D}^{2}\;+\sigma_{S}^{2}\;+\sigma_{E}^{2}},$$

such that

VarT +VarD+VarS +VarE =1.

Under this normalization, VarT is numerically identical to the ICC. ICC estimates were reported with bootstrapped 95% confidence intervals based on 1,000 resamples. Path diagrams (Fig. 2) were visualized in Ωnyx (Von Oertzen et al., 2015). To assess the potential influence of age on reliability estimates, ICED models were re-estimated using age-residualized metabolite values (Supplementary Table S2; Supplementary Fig. S1). As an additional sensitivity analysis (Supplementary Methods), the models were additionally re-estimated after excluding two participants with low voxel-placement consistency, defined as a mean Dice coefficient < 0.70 across pairwise comparisons (Zijdenbos et al., 1994; Zou et al., 2004) (Supplementary Table S3; Supplementary Fig. S2).

**Fig. 2.**
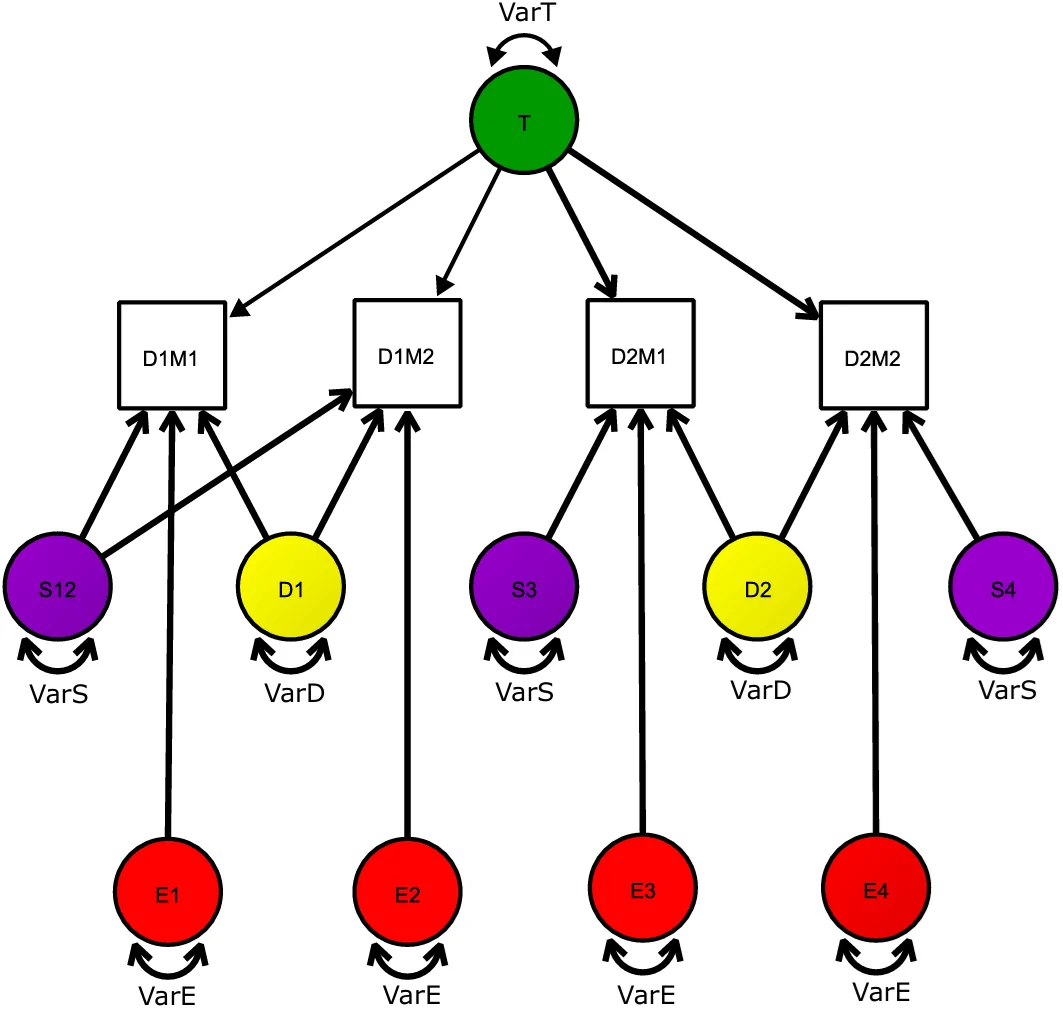
Path diagram of the structural equation model. The diagram illustrates the intra-class effect decomposition (ICED) model applied to our repeated-measures design. Each participant was scanned four times: twice on day 1 with 128 transients (D1M1 and D1M2) and twice on day 2 with 256 transients (D2M1) and 128 transients (D2M2). A saturated mean structure was used. Abbreviations: VarT, true-score variance; VarS, session-specific variance; VarD, day-specific variance; VarE, residual error variance.

### 2.8 Acquisition and measurement characteristics

#### 2.8.1 Tissue composition, spectral quality, and CRLB values

To examine whether tissue composition, spectral quality metrics, and CRLB values differed across measurement occasions, we fitted separate linear mixed-effects models for GM, WM, and CSF fractions, SNR, LW, and metabolite-specific CRLB values. These analyses were used to assess whether acquisition characteristics and data-quality metrics were stable across measurements.

#### 2.8.2 Metabolite concentrations

To examine whether metabolite concentrations differed systematically across measurement occasions, separate linear mixed-effects models were fitted for each metabolite. These analyses complemented the primary test–retest analyses by testing whether the group mean metabolite concentration differed across measurement occasions, which is not directly addressed by the CV or ICED analyses. Each model included measurement occasion as a fixed effect and participant as a grouping variable for a random intercept. Models were fitted using the nlme package in R (Pinheiro et al., 2025) and estimated with restricted maximum likelihood and listwise deletion for missing data. Significant main effects were followed up with Tukey-adjusted pairwise comparisons.

#### 2.8.3 Exploratory analysis of voxel overlap

Voxel-placement consistency was assessed using Dice coefficients (Crum et al., 2006) computed between voxel masks from the four single-voxel MRS acquisitions, as differences in voxel placement can affect measured metabolite concentrations (Bai et al., 2017). Dice coefficients were computed for all pairwise combinations of the four measurements for each participant, and group-level summaries are reported in Supplementary Table S13.

To examine whether lower voxel overlap was associated with poorer agreement between repeated metabolite measurements, we computed absolute between-measurement differences in metabolite concentration for the corresponding measurement pairs. Exploratory linear mixed-effects models were then used to test whether Dice coefficients predicted these absolute pairwise metabolite differences. Full details of the Dice computation, model specification, and sensitivity analysis are provided in Supplementary Methods section 1.4.

## 3 Results

### 3.1 Absolute reliability of metabolite estimates

Across metabolites, mean CVs ranged from approximately 4% to 17% (Fig. 3; Supplementary Table S1). Thus, mean CVs were below 20% for all metabolites and below 10% for tNAA, tCho, tCr, Glx, Glu, and mI.

**Fig. 3.**
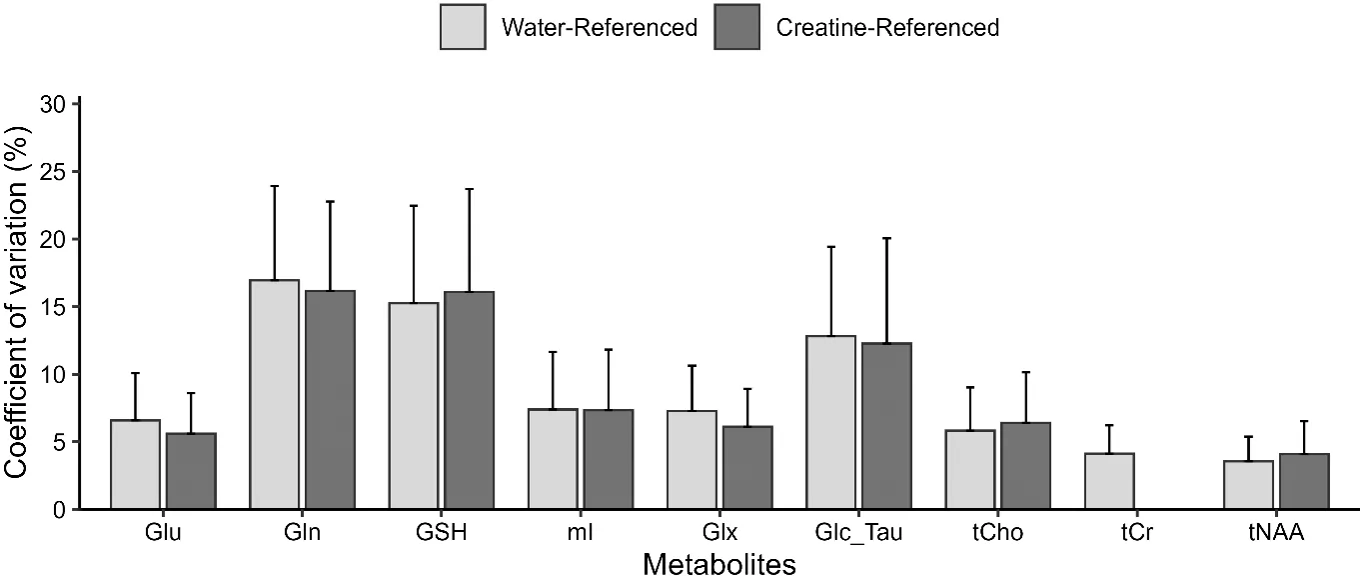
Coefficient of variation.

### 3.2 Relative reliability of metabolite estimates

To evaluate relative reliability, we applied ICED to partition variance in metabolite estimates into VarT, VarD, VarS, and VarE. This analysis revealed considerable heterogeneity across metabolites (Table 1, Fig. 4).

**Fig. 4.**
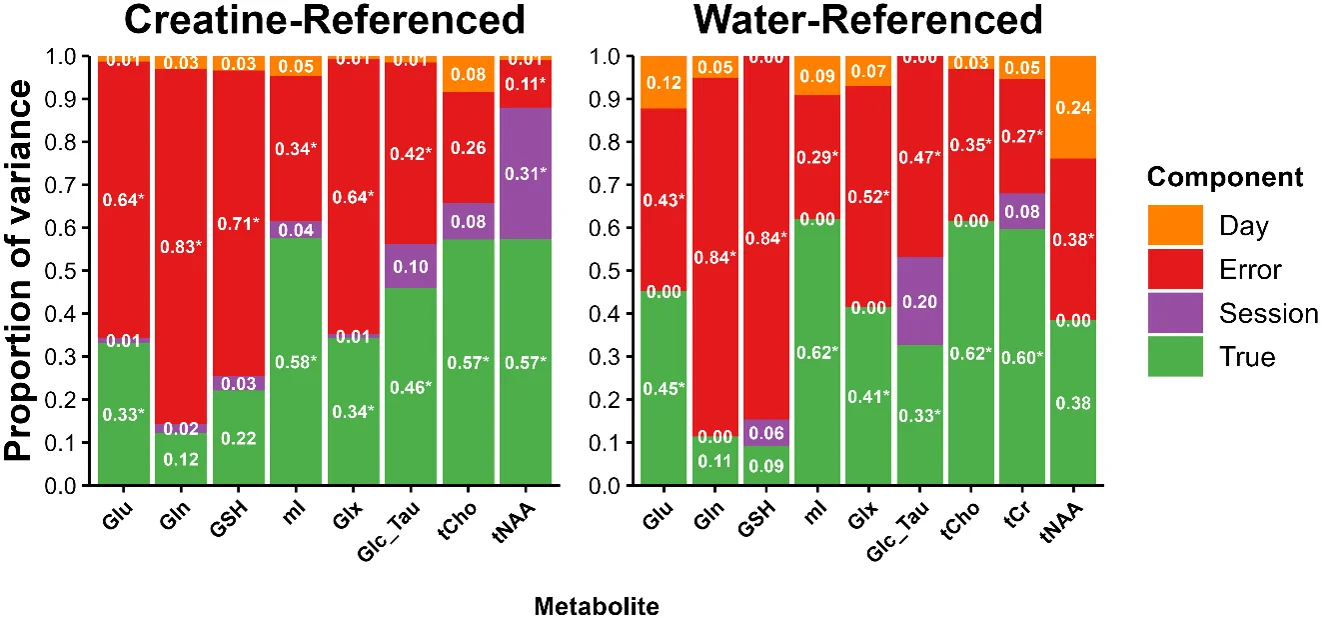
Variance components. Intra-class effect decomposition of hippocampal metabolite estimates. Bars show the proportion of total variance attributable to true-score variance (VarT), day effects (VarD), session effects (VarS), and residual error (VarE). Asterisks indicate significant variance components (p < .05).

**Table 1.** Variance estimates and relative reliability measures.

Reference	Metabolite	RMSEA	VarT	VarD	VarS	VarE	ICC	95% CI
Creatine	Glu	0.20	0.33*	0.01	0.01	0.64*	0.33	0.01-0.65
	Gln	0.06	0.12	0.03	0.02	0.83*	0.12	0.02-0.28
	GSH	0.23	0.22	0.03	0.03	0.71*	0.22	0.04-0.43
	mI	0.16	0.58*	0.05	0.04	0.34*	0.58	0.28-0.74
	Glx	0.02	0.34*	0.01	0.01	0.64*	0.34	0.02-0.59
	Glc_Tau	0.32	0.46*	0.01	0.10	0.42*	0.46	0.16-0.65
	tCho	0.29	0.57*	0.08	0.08	0.26	0.57	0.21-0.76
	tNAA	0.28	0.57*	0.01	0.31*	0.11*	0.57	0.02-0.80
Water	Glu	0.00	0.45*	0.12	0.00	0.43*	0.45	0.14-0.68
	Gln	0.13	0.11	0.05	0.00	0.84*	0.11	0.00-0.31
	GSH	0.23	0.09	0.00	0.06	0.84*	0.09	0.00-0.31
	mI	0.16	0.62*	0.09	0.00	0.29*	0.62	0.18-0.81
	Glx	0.26	0.41*	0.07	0.00	0.52*	0.41	0.15-0.62
	Glc_Tau	0.26	0.33*	0.00	0.20	0.47*	0.33	0.11-0.49
	tCho	0.10	0.62*	0.03	0.00	0.35*	0.62	0.25-0.79
	tCr	0.00	0.60*	0.05	0.08	0.27*	0.60	0.28-0.77
	tNAA	0.09	0.38	0.24	0.00	0.38*	0.38	0.00-0.73

* Indicates a significant chi-square difference from the null model (p < .05 for VarT, VarD, and VarS) or a significant Wald test (p < .05 for VarE).

Abbreviations: VarT, true-score variance; VarD, day-specific variance; VarS, session-specific variance; VarE, residual error variance; ICC, intraclass correlation coefficient; CI, confidence interval; RMSEA, root mean squared error of approximation.

For the creatine-referenced data, VarT ranged from 0.12 to 0.58. VarT was significant for Glu, mI, Glx, Glc_Tau, tCho, and tNAA, but not for Gln or GSH. For the water-referenced data, VarT ranged from 0.09 to 0.62. VarT was significant for Glu, mI, Glx, Glc_Tau, tCho, and tCr, but not for Gln, GSH, or tNAA.

Across both quantification methods, day-specific variance components were small and none were significant. Session-specific variance components were also small, except for creatine-referenced tNAA, which showed a significant VarS component.

### 3.3 Acquisition and measurement characteristics

#### 3.3.1 Tissue composition and measurement quality

Linear mixed-effects models revealed no significant difference across measurement occasion for GM (F(3, 49) = 1.80, p = .16), WM (F(3, 49) = 1.57, p = .21), or CSF (F(3, 49) = 0.53, p = .66), indicating stable tissue composition across measurements (Supplementary Table S4). Average CVs were low for GM, moderate for WM, and higher for CSF (Table 2).

**Table 2.** Tissue fractions and spectral quality metrics for each session.

	D1M1	D1M2	D2M1	D2M2	CV
GM	0.68 ± 0.05 a	0.69 ± 0.05 a	0.68 ± 0.06 a	0.66 ± 0.05 a	0.05 ± 0.03
WM	0.28 ± 0.05 a	0.27 ± 0.04 a	0.28 ± 0.06 a	0.29 ± 0.05 a	0.11 ± 0.06
CSF	0.04 ± 0.03 a	0.04 ± 0.03 a	0.04 ± 0.03 a	0.04 ± 0.03 a	0.22 ± 0.19
SNR	20.53 ± 2.86 b	19.16 ± 4.98 b	26.37 ± 3.20 a	20.16 ± 3.08 b	0.19 ± 0.10
LW	7.62 ± 0.80 a	7.44 ± 0.54 a	7.80 ± 1.21 a	7.72 ± 0.89 a	0.05 ± 0.04

Values are means ± SD. Compact letter displays indicate pairwise differences between sessions: sessions sharing the same letter do not differ significantly, whereas different letters (a or b) denote significant differences (p < 0.05).

Abbreviations: GM, gray matter; WM, white matter; CSF, cerebrospinal fluid; SNR, signal-to-noise ratio; LW, linewidth; CV, coefficient of variation.

For spectral quality, SNR differed significantly across measurement occasion, F(3, 49) = 36.71, p < .001 (Supplementary Table S5). Post hoc pair-wise comparisons showed that SNR was higher at Day 2 Measurement 1 (D2M1) than at all other measurements (all ps < .001), reflecting the greater number of transients (256 vs. 128). No other pairwise differences were significant (all ps > .90). LW did not differ across measurement occasions, F(3, 49) = 0.41, p = .75. CVs were moderate for SNR and low for LW (Table 2); however, differences in the number of transients across measurements limit the interpretability of the CV of SNR.

#### 3.3.2 CRLB values

Linear mixed-effects models tested for differences in CRLB values across measurement occasions (Fig. 5; Supplementary Table S7). Omnibus tests indicated significant differences across measurement occasions for all metabolites (Supplementary Table S6). At D2M1 (256 transients), CRLB values were generally lower than at least one other measurement for most metabolites, consistent with the larger number of transients in this acquisition (Supplementary Table S8).

**Fig. 5.**
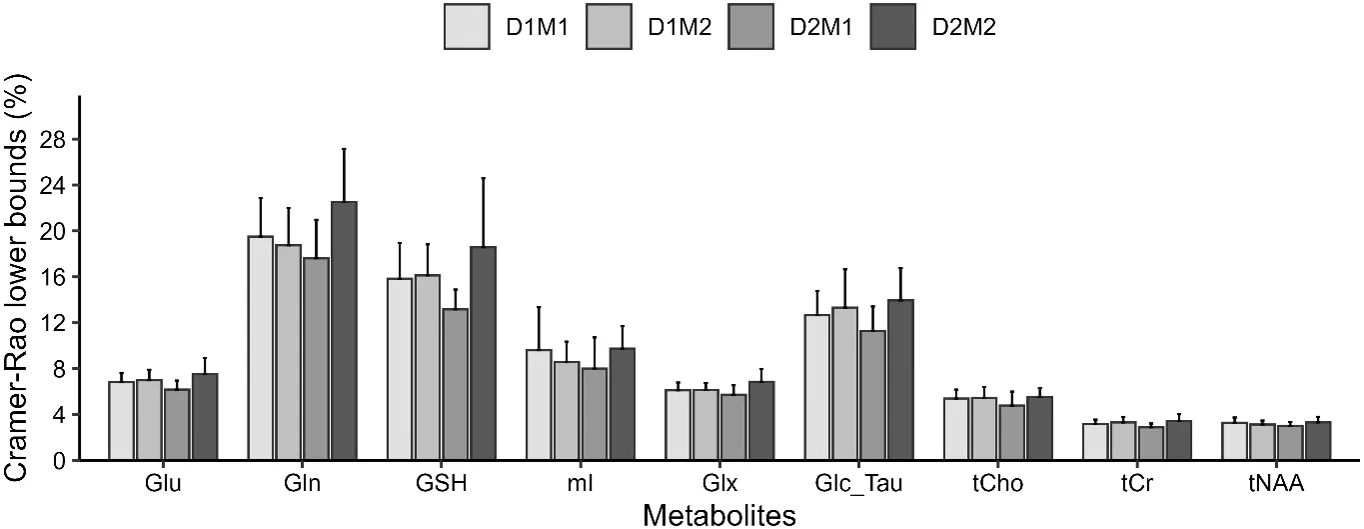
CRLB values for each metabolite and measurement. CRLB values are shown for each measurement. Values are mean ± SD. Abbreviations: D1M1, day 1 measurement 1; D1M2, day 1 measurement 2; D2M1, day 2 measurement 1; D2M2, day 2 measurement 2.

#### 3.3.3 Mean metabolite concentrations across measurements

Mean metabolite concentrations differed across measurement occasions for a small subset of metabolites (Fig. 6; Supplementary Tables S9-S12). Significant omnibus effects were observed for water-referenced Gln, Glu, and Glx, and for creatine-referenced Glx and tCho. Tukey-adjusted pairwise comparisons indicated lower concentrations at later measurement occasions for water-referenced Gln, Glu, and Glx, and for creatine-referenced Glx; however, no pairwise contrast reached significance for creatine-referenced tCho despite the significant omnibus effect.

**Fig. 6.**
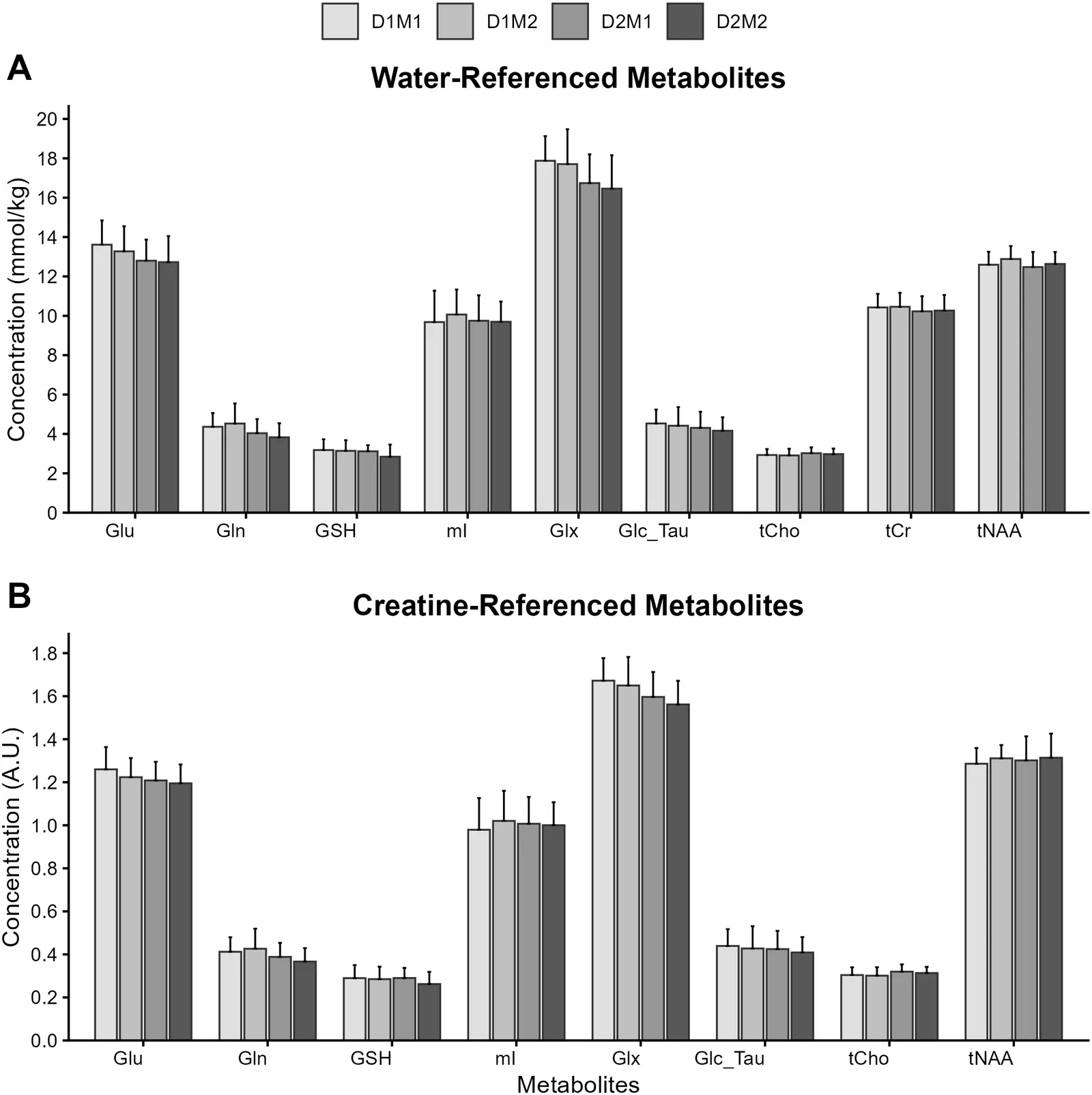
Mean metabolite concentrations. Mean metabolite concentrations are shown for each session. (A) Water-referenced, tissue-corrected metabolites. (B) Metabolites referenced to total creatine. Error bars represent between-participant standard deviations.

### 3.4 Exploratory voxel-overlap analyses

Exploratory linear mixed-effects models showed a negative association between voxel overlap and absolute between-measurement differences for water-referenced tNAA in the full sample, whereas no significant associations were observed for the remaining metabolites (Supplementary Table S14; Supplementary Fig. S3). Re-estimation of these models after excluding two participants with low voxel-placement consistency showed that no significant associations were observed (Supplementary Table S15; Supplementary Fig. S4).

## 4 Discussion

This study applied the ICED framework to quantify sources of variance underlying test–retest reliability of hippocampal metabolite estimates acquired with single-voxel semi-LASER MRS at 3 T. By partitioning total variance into true-score, day-specific, session-specific, and residual components, we aimed to disentangle stable interindividual differences from other sources of variance. Across metabolites, relative reliability ranged from poor to good, with VarT accounting for a variable proportion of total variance across metabolites. All day-level components were small and nonsignificant, and session-level components were nonsignificant except for a significant effect for tNAA (creatine-referenced). Together, these results suggest that most unreliability in hippocampal MRS reflects variance not captured by identifiable factors associated with differences between days or the number of transients (and resulting SNR differences).

The true-score variance component (VarT) reflects stable between-person differences across all measurements and is equivalent to the ICC. A large VarT indicates strong rank-order stability, whereas a small VarT indicates that stable between-person differences account for a smaller proportion of total variance. This distinction is particularly relevant as neuroscience and psychiatry increasingly adopt dimensional frameworks aimed at identifying neurochemical markers of individual variability in cognition and psychopathology (Cuthbert & Insel, 2013; Insel et al., 2010; Marek et al., 2022; Paulus & Thompson, 2019). Such approaches assume that individual metabolite levels reflect stable biological traits; hence, limited VarT constrains the interpretability of brain–behavior associations and the potential for MRS to serve as a reliable biomarker (Elliott et al., 2020).

In the present data, VarT accounted for roughly 30–60% of total variance for most metabolites, equivalent to ICC values of 0.30–0.60 and corresponding to relative reliability estimates spanning poor, fair, and good ranges. This is broadly consistent with previous hippocampal findings obtained with a LASER sequence at 3 T with 768 transients, where ICCs ranged from 0.38 to 0.75 (Allaïli et al., 2015). Importantly, broadly comparable ICC magnitudes were observed here with substantially fewer transients. Comparable values have also been reported for other limbic and subcortical regions with semi-LASER at 3 T (Nacewicz et al., 2012; Shyu et al., 2021), whereas cortical voxels such as motor, frontal, and posterior cingulate typically show higher relative reliability (approximately 0.6–0.9) (Eftekhari et al., 2025; Macdonald et al., 2025; McGuire et al., 2017).

VarT proportions were broadly similar across referencing methods for most metabolites, although some differences were observed. For example, VarT was slightly higher for water–referenced mI, tCho, Glu, and Glx than for the corresponding tCr-referenced estimates, whereas the opposite pattern was observed for GSH, Glc+Tau, and tNAA. The clearest reference-dependent pattern was observed for tNAA, widely used as a marker of neuronal integrity and metabolism (Baslow, 2000; Moffett & Namboodiri, 2006). Water–referenced tNAA showed nonsignificant VarT both in the main analysis and after excluding participants with low voxel overlap, whereas creatine-referenced tNAA showed significant VarT only in the main analysis. One possible explanation for the lower water–referenced VarT is that correction for voxel composition and relaxation effects reduced detectable between-person variability by removing systematic but non-metabolite sources of variance. Since tNAA is the most prominent spectral peak and showed high absolute reliability (low CVs), the relatively low water-referenced relative reliability is therefore more likely due to limited true variability between participants than to measurement noise. However, the creatine-referenced VarT finding should also be interpreted cautiously, because it was no longer significant after excluding participants with low voxel overlap.

The session-specific variance component (VarS) reflects systematic variability linked to a particular measurement session; in the present design, this primarily reflects the unequal number of transients. A significant VarS therefore indicates that acquisition length affected the relative-reliability structure, whereas a small or nonsignificant VarS suggests little such effect. The 256-transient session yielded higher SNR but did not translate into higher relative reliability. This may indicate that improvements in signal quality affected participants similarly, or that longer acquisition introduced additional random variability (e.g., due to motion or physiological drift). Thus, the present semi-LASER findings do not provide evidence that the reliability benefit of extended acquisition length reported by Allaïli et al. (2015) generalizes straightforwardly to semi-LASER hippocampal MRS.

Only creatine-referenced tNAA showed a significant VarS, accounting for 31% of total variance despite no detectable mean session difference in the linear mixed model. This suggests that acquisition length affected the relative-reliability structure for creatine-referenced tNAA, even though it did not detectably change mean estimates. For creatine-referenced tNAA, this finding underscores the importance of consistent acquisition lengths in repeated-measures designs that depend on stable rank-order differences, such as longitudinal or multisite studies.

The residual variance component (VarE) captures variability unexplained by the model, including both random noise and systematic but unmodeled sources such as subtle voxel misalignment, participant motion, differences in shimming quality, physiological noise, and frequency drift (Andronesi et al., 2021; Hui et al., 2021; Juchem et al., 2021). VarE accounted for 11-84% of total variance across metabolites, with several estimates above 50%, showing that unmodeled variability remained a major factor limiting relative reliability for several metabolites.

The day-specific variance component (VarD) reflects systematic variability between measurement days, such as differences in participant state, scanner calibration, or environmental conditions that are stable within a day but vary across days. In the present data, the VarD components were generally small and not significant for any metabolite, indicating that day-specific influences on relative reliability were minimal.

Absolute reliability was generally high, with low CVs for several metabolites, and was comparable to values reported by Bednařík et al. (2015) and to semi-LASER results in cortical and cerebellar regions at 3 T (Deelchand et al., 2015; Terpstra et al., 2016). These values were also generally lower and more consistent across metabolites than in other hippocampal studies (Allaïli et al., 2015; Gajdošík et al., 2021; Venkatraman et al., 2006; Zhang et al., 2018). Nevertheless, relative reliability was generally poor or fair, with only water–referenced mI, tCho, and tCr reaching good ICCs. Thus, low within-person variability did not necessarily translate into strong rank-order stability. The opposite pattern has also been reported in the nucleus accumbens (Liu et al., 2017), where higher ICCs (0.77–0.88) were observed despite larger CVs (8–14%), further illustrating that high absolute reliability and high relative reliability do not necessarily coincide.

The dominance of residual variance for most metabolites indicates that a large proportion of total variance was not attributed to stable between-person differences, day-level effects, or session-specific effects. Importantly, this does not necessarily imply that measurement error was large in absolute terms. A large VarE proportion may reflect unmodeled measurement variability, limited true between-person variability, or both. Several measures were implemented to minimize measurement-related sources of variability, including a semi-LASER sequence with FAST(EST)MAP shimming and interleaved outer-volume and water suppression, foam padding to reduce head motion, voxel placement performed by the same trained operator for all measurements, and spectral modeling in Osprey and LCModel to ensure standardized preprocessing and sequence-specific basis fitting. These optimizations yielded narrow linewidths and high spectral quality, yet VarE still accounted for a substantial portion of total variance. This suggests that remaining unreliability may reflect subtle measurement-related sources, such as within-scan motion, dynamic changes in B0 homogeneity, frequency drift, or residual preprocessing and quantification variability, as well as limited stable interindividual variability for some metabolites. The use of a 20-channel head coil may also have limited SNR relative to newer 32-channel or 48-channel coils. Although higher SNR does not necessarily improve relative reliability, lower SNR could contribute to fitting uncertainty and residual measurement variability, and may therefore have contributed to VarE for some metabolites.

Because voxel-placement differences are one plausible contributor to residual variance, we explored whether voxel overlap was associated with pairwise differences in metabolite estimates. In the full sample, lower voxel overlap was associated with larger pairwise differences in metabolite estimates only for water-referenced tNAA, whereas no significant associations were observed for the other metabolites. After excluding participants with low voxel overlap, no associations between voxel overlap and pairwise differences in metabolite estimates remained significant. This suggests that voxel overlap may matter when overlap is unusually poor, but it does not support voxel-placement inconsistency as a general explanation for metabolite variability. Consistent with this interpretation, the ICED sensitivity analysis excluding low-overlap participants did not remove the broader pattern of residual variance, indicating that VarE is unlikely to be explained by voxel-placement differences alone.

Future work should therefore target multiple sources of residual error, including voxel-placement variability, within-scan motion, dynamic B0 changes, frequency drift, and residual preprocessing or quantification variability. Relevant approaches include higher-sensitivity receiver arrays, automated voxel placement (Bishop et al., 2023; Park et al., 2018), real-time motion correction (Adanyeguh et al., 2024; Saleh et al., 2020), dynamic shimming (Öz et al., 2021), and improved preprocessing and quantification pipelines (Near et al., 2021). However, methodological refinements may not fully resolve low relative reliability if stable between-person variability is limited in the population being studied.

In addition to acquisition- and analysis-related factors, sample characteristics may also contribute to reliability differences across studies. The present sample was modest in size (n = 19), although larger than in most previous hippocampal reproducibility studies and comparable to prior ICED applications (Anand et al., 2022; Wenger et al., 2022). The modest sample size likely reduced the precision of the estimated variance components and contributed to wider confidence intervals, and the ICED results should therefore be interpreted with this in mind. The sample also covered a relatively broad age range, which could partly explain discrepancies in reliability estimates relative to other studies, alongside differences in acquisition parameters and modeling choices. To examine whether age contributed to the reliability estimates, we repeated the ICED analysis using age-residualized metabolite values. Overall patterns were similar to the main analysis, although VarT estimates decreased for some metabolites, suggesting that age-related variability may have contributed to stable between-person differences in those cases. Future studies could extend this approach by applying comparable repeated-measures designs across acquisition protocols and study populations, including clinical or more heterogeneous samples. This would help determine whether low relative reliability mainly reflects residual error variance, which should decrease under improved acquisition or processing conditions, or limited stable between-person variability, which may differ across populations.

Finally, reliability estimates may also depend on the quantification approach. Here, preprocessing was performed with Osprey, which enables transparent and reproducible processing. Quantification was conducted using LCModel, a well-validated method that facilitates comparison with prior MRS literature. Nevertheless, alternative modeling strategies (e.g., Clarke et al., 2021; Wilson et al., 2011) may yield partly divergent estimates due to differences in baseline handling, prior knowledge implementation, or fitting algorithms.

### 4.1 Conclusion

In summary, this study demonstrates that the ICED framework can disentangle distinct sources of measurement variance in hippocampal MRS, providing a more detailed account of reliability than conventional single-value metrics. Despite good spectral quality and high absolute reliability for several metabolites, relative reliability was generally low to fair. No creatine-referenced metabolites reached good relative reliability, whereas only water–referenced mI, tCho, and tCr did. Consequently, relative reliability was often below the good-to-excellent range typically preferred for studies relying on stable between-person differences (Cicchetti, 1994; Koo & Li, 2016). These findings suggest that hippocampal MRS at 3 T may be suitable for studies focused on group-level or mean-level metabolite estimates but highlight limitations for individual-difference, correlational, or biomarker applications that depend on stable rank-order differences between participants. Continued methodological refinement will be important for reducing residual variance and improving the use of hippocampal MRS as a quantitative measure of hippocampal function and integrity.

## Supplementary Material

Supplementary Material

## Data Availability

The data underlying this study cannot be made publicly available because ethical approval does not permit public archiving of pseudonymized study data. Researchers seeking access to the data should contact Goran Papenberg (goran.papenberg@ki.se) or Martin Nilsson (martin.nilsson@ki.se). Requestors must meet the following conditions for data access: completion of a formal data sharing agreement and ethical approval. Analysis code is available at https://osf.io/7x52z/overview
