# Supplementary material for: Assessing the reliability of hippocampal single-voxel proton magnetic resonance spectroscopy through intra-class effect decomposition
Source: Imaging Neurosci (Camb). 2026 Oct 16;4:IMAG.a.1401. doi: 10.1162/IMAG.a.1401 (PMC13681051; doi:10.1162/IMAG.a.1401)
Supplement: Supplementary file 1 [file IMAG.a.1401_supp.pdf]

Supplementary Information for: Assessing the reliability of hippocampal single-voxel proton magnetic resonance spectroscopy through intra-class effect decomposition

This file contains supplementary figures and tables.

1 Supplementary Methods

1.1 Absolute reliability

Within-participant coefficients of variation were calculated across the four acquisitions for each metabolite and quantification scheme and summarized across participants as means ± SD.

1.2 Age-residualized relative reliability analyses

Age-residualized metabolite values were obtained from residuals of regressions of metabolite values on age. These residualized values were then used in the same intra-class effect decomposition framework as the primary reliability analyses.

1.3 ICED sensitivity analysis excluding low-overlap participants

As an additional sensitivity analysis, the ICED models were rerun after excluding participants with low voxel-placement consistency, defined as a mean Dice coefficient < 0.70 across all pairwise voxel comparisons. This analysis was performed to examine whether participants with comparatively low spatial overlap disproportionately contributed to the estimated reliability structure. The ICED model specification was otherwise identical to the main analysis.

1.4 Exploratory Voxel-Overlap Analyses

Voxel-placement consistency was assessed using Dice coefficients (Crum et al., 2006) computed between voxel masks from the four single-voxel MRS acquisitions, as differences in voxel placement can affect measured metabolite concentrations (Bai et

al., 2017). The Dice coefficient between two voxel masks A and B was defined as $\mathrm{Dice}(A,B) = \frac{2|A \cap B|}{|A|+|B|}$. Voxel masks were generated by Osprey using SPM12 normalization to MNI space (Ashburner & Friston, 2005). Note that, because Dice coefficients were computed after normalization to MNI space, these values may include effects of normalization-related deformation or interpolation in addition to differences in native-space voxel placement (Bai et al., 2017). Dice coefficients were computed for all pairwise combinations of the four measurements for each participant.

For the corresponding measurement pairs, absolute between-measurement differences in metabolite concentration were computed. To evaluate whether spatial overlap between voxel masks was associated with differences in metabolite concentration estimates, separate linear mixed-effects models were fitted for each metabolite, with absolute between-measurement differences as the outcome, Dice coefficient as a fixed effect, and participant as a grouping variable for a random intercept. Dice coefficients were grand-mean centered prior to analysis.

A sensitivity analysis was performed in which the linear mixed-effects models were repeated after excluding participants with low voxel-placement consistency, defined as a mean Dice coefficient < 0.70 across all pairwise comparisons. This threshold was used as a pragmatic criterion to exclude cases with comparatively low spatial overlap, informed by image-segmentation literature in which Dice values > 0.70 have been interpreted as indicating good spatial overlap (Zijdenbos et al., 1994; Zou et al., 2004).

2 Supplementary Results

2.1 Absolute reliability of metabolite estimates

***Table S1**. Coefficient of variation*

Metabolite	Creatine-referenced	Water-referenced
Gln	16.2±6.6	16.9±7.0
Glu	5.6±3.0	6.6±3.5
GSH	16.1±7.6	15.3±7.2
mI	7.3±4.5	7.4±4.3
tCho	6.4±3.7	5.8±3.2
tCr		4.1±2.1
tNAA	4.1±2.4	3.6±1.8
Glx	6.1±2.8	7.3±3.4
Glc_Tau	12.3±7.8	12.8±6.6

Note. Values are means ± SD.

2.2 Relative Reliability

2.3 ICED analysis using age-residualized metabolite values

***Figure S1**. Age-residualized variance estimates and reliability measures*

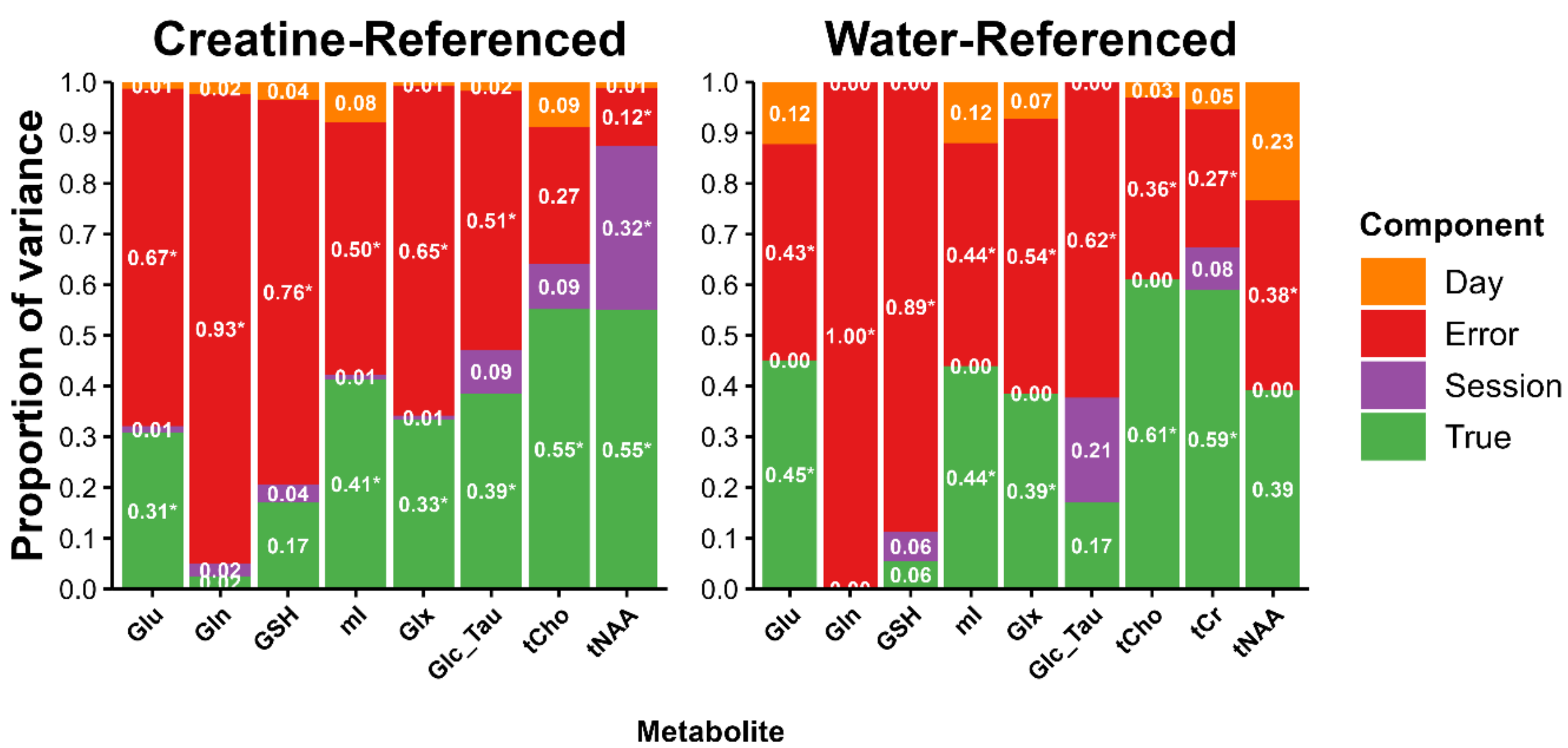

Note. Intra-class effect decomposition of age-residualized hippocampal metabolite estimates. Bars show the proportion of total variance attributable to true between-person differences (VarT), day effects (VarD), session effects (VarS), and residual error (VarE). Asterisks indicate significant variance

components ($p < 0.05$). VarT = true score variance, VarS = session-specific variance, VarD = day-specific variance, VarE = residual error variance.

Table S2. *Age-residualized variance estimates and reliability measures*

Reference	Metabolite	rmsea	VarT	VarD	VarS	VarE	ICC	95% CI
Creatine	Glu	0.19	0.31*	0.01	0.01	0.67*	0.31	0.01-0.64
	Gln	0.12	0.02	0.02	0.02	0.93*	0.02	0.02-0.04
	GSH	0.23	0.17	0.04	0.04	0.76*	0.17	0.04-0.38
	mI	0.10	0.41*	0.08	0.01	0.50*	0.41	0.07-0.63
	Glx	0.07	0.33*	0.01	0.01	0.65*	0.33	0.01-0.59
	Glc_Tau	0.33	0.39*	0.02	0.09	0.51*	0.39	0.04-0.61
	tCho	0.29	0.55*	0.09	0.09	0.27	0.55	0.19-0.71
	tNAA	0.25	0.55*	0.01	0.32*	0.12*	0.55	0.05-0.77
Water	Glu	0.00	0.45*	0.12	0.00	0.43*	0.45	0.10-0.66
	Gln	0.18	0.00	0.00	0.00	1.00*	0.00	0.00-0.03
	GSH	0.24	0.06	0.00	0.06	0.89*	0.06	0.00-0.27
	mI	0.10	0.44*	0.12	0.00	0.44*	0.44	0.00-0.73
	Glx	0.30	0.39*	0.07	0.00	0.54*	0.39	0.12-0.58
	Glc_Tau	0.34	0.17	0.00	0.21	0.62*	0.17	0.00-0.42
	tCho	0.09	0.61*	0.03	0.00	0.36*	0.61	0.27-0.78
	tCr	0.06	0.59*	0.05	0.08	0.27*	0.59	0.25-0.78
	tNAA	0.12	0.39	0.23	0.00	0.38*	0.39	0.00-0.72

Note. Metabolite values were age-residualized using residuals from regressions of metabolite values on age. Indicates a significant chi-square difference from the null model ($P < .05$ for VarT, VarD, and VarS) or a significant Wald test ($P < .05$ for VarE). VarT = true score variance, VarS = session-specific variance, VarD = day-specific variance, VarE = residual error variance, RMSEA = root mean squared error of approximation.

We repeated the ICED analysis using age-residualized metabolite estimates (Table S11; Figure S3). Overall patterns were similar to the main analysis. For the creatine-referenced data, VarT remained significant for Glu, mI, Glx, Glc_Tau, tCho, and tNAA, but not for Gln or GSH. For the water-referenced data, VarT remained significant for Glu, mI, Glx, tCho, and tCr, but not for Gln, GSH, Glc_Tau, or tNAA. Day-specific

variance components were not significant, and only creatine-referenced tNAA showed a significant session-specific variance component.

2.3.1 ICED sensitivity analysis excluding participants with low voxel overlap

Figure S2*. Variance estimates and reliability measures after excluding two participants with low voxel overlap*

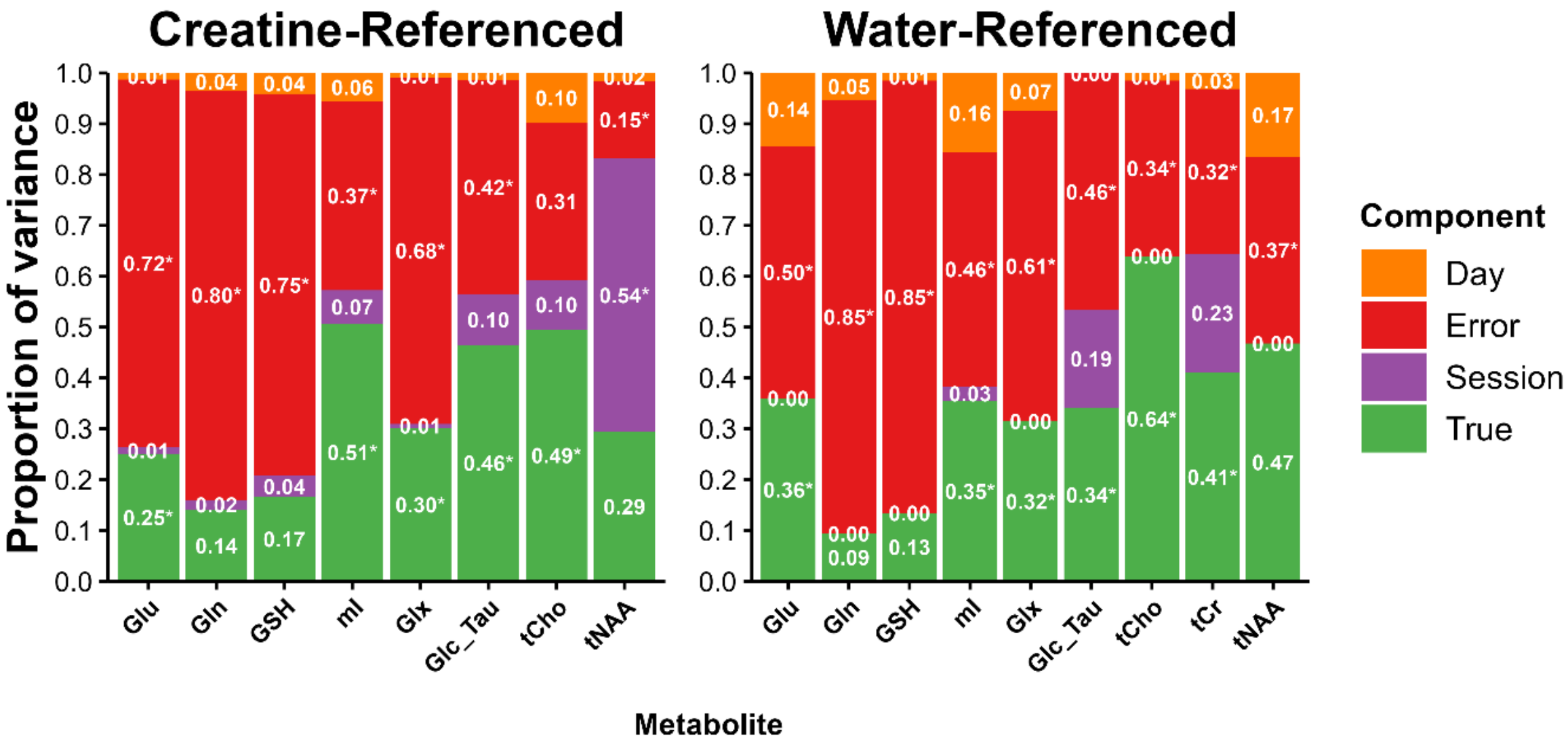

Note. Intra-class effect decomposition of hippocampal metabolite estimates after excluding two participants with mean Dice coefficient < 0.7 (N=17). Bars show the proportion of total variance attributable to true between-person differences (VarT), day effects (VarD), session effects (VarS), and residual error (VarE). Asterisks indicate significant variance components ($p < 0.05$). VarT = true score variance, VarS = session-specific variance, VarD = day-specific variance, VarE = residual error variance.

Table S3. *Variance estimates and reliability measures after excluding two participants with low voxel overlap*

Reference	Metabolite	rmsea	VarT	VarD	VarS	VarE	ICC	95% CI
Creatine	Glu	0.19	0.25*	0.01	0.01	0.72*	0.25	0.01-0.60
	Gln	0.06	0.14	0.04	0.02	0.80*	0.14	0.02-0.30
	GSH	0.16	0.17	0.04	0.04	0.75*	0.17	0.04-0.41
	mI	0.26	0.51*	0.06	0.07	0.37*	0.51	0.20-0.73
	Glx	0.10	0.30*	0.01	0.01	0.68*	0.30	0.01-0.56
	Glc_Tau	0.35	0.46*	0.01	0.10	0.42*	0.46	0.18-0.66
	tCho	0.28	0.49*	0.10	0.10	0.31	0.49	0.13-0.72
	tNAA	0.21	0.29	0.02	0.54*	0.15*	0.29	0.01-0.65
Water	Glu	0.08	0.36*	0.14	0.00	0.50*	0.36	0.04-0.60

Reference	Metabolite	rmsea	VarT	VarD	VarS	VarE	ICC	95% CI
	Gln	0.14	0.09	0.05	0.00	0.85*	0.09	0.00-0.25
	GSH	0.20	0.13	0.01	0.00	0.85*	0.13	0.00-0.38
	mI	0.18	0.35*	0.16	0.03	0.46*	0.35	0.11-0.56
	Glx	0.28	0.32*	0.07	0.00	0.61*	0.32	0.00-0.50
	Glc_Tau	0.29	0.34*	0.00	0.19	0.46*	0.34	0.13-0.52
	tCho	0.07	0.64*	0.01	0.00	0.34*	0.64	0.26-0.81
	tCr	0.17	0.41*	0.03	0.23	0.32*	0.41	0.11-0.60
	tNAA	0.22	0.47	0.17	0.00	0.37*	0.47	0.00-0.80

Note. Intra-class effect decomposition of hippocampal metabolite estimates after excluding two participants with mean Dice coefficient < 0.7 (N = 17). Asterisk indicates a significant chi-square difference from the null model (P < .05 for VarT, VarD, and VarS) or a significant Wald test (P < .05 for VarE). VarT = true score variance, VarS = session-specific variance, VarD = day-specific variance, VarE = residual error variance, RMSEA = root mean squared error of approximation.

2.4 Acquisition and Measurement Characteristics

2.4.1 Tissue composition, spectral quality, and CRLB values

Table S4*. Tissue composition and spectral quality metrics omnibus test*

	F-value	p-value
GM	F(3, 49) = 1.80	0.159
WM	F(3, 49) = 1.57	0.209
CSF	F(3, 49) = 0.53	0.662
SNR	F(3, 49) = 36.71	<.001
LW	F(3, 49) = 0.41	0.749

Note. Omnibus tests are from linear mixed-effects models with Measurement as fixed effect and participant-specific random intercepts. GM = gray matter; WM = white matter; CSF = cerebrospinal fluid; SNR = signal-to-noise ratio; LW = linewidth.

Table S5*. Signal-to-noise ratio (SNR) pairwise comparisons*

Contrast	Estimate	SE	t-stat	p-value
D1M1 vs D1M2	0.01	0.72	0.01	0.999
D1M1 vs D2M1	-5.80	0.69	-8.35	<.001
D1M1 vs D2M2	0.42	0.68	0.62	0.927

Contrast	Estimate	SE	t-stat	p-value
D1M2 vs D2M1	-5.81	0.72	-8.05	<.001
D1M2 vs D2M2	0.41	0.71	0.58	0.938
D2M1 vs D2M2	6.22	0.68	9.12	<.001

Note. Pairwise comparisons are Tukey-adjusted estimated marginal mean contrasts from the linear mixed-effects model for signal-to-noise ratio. SE = standard error.

2.5 CRLB

Table S6. *CRLBs for each metabolite and session*

Metabolite	D1M1	D1M2	D2M1	D2M2
GSH	15.8 ± 3.1 ab	16.1 ± 2.7 ab	13.2 ± 1.7 a	18.6 ± 6.0 b
Glc_Tau	12.7 ± 2.1 ab	13.3 ± 3.3 ab	11.3 ± 2.1 a	13.9 ± 2.8 b
Gln	19.5 ± 3.4 ab	18.8 ± 3.2 a	17.6 ± 3.3 a	22.5 ± 4.6 b
Glu	6.8 ± 0.8 ab	7.0 ± 0.9 ac	6.2 ± 0.8 b	7.5 ± 1.4 c
Glx	6.1 ± 0.7 a	6.1 ± 0.6 a	5.7 ± 0.8 a	6.8 ± 1.1 b
mI	9.6 ± 3.7 a	8.6 ± 1.8 a	8.0 ± 2.7 a	9.7 ± 2.0 a
tCho	5.4 ± 0.8 ab	5.4 ± 1.0 a	4.8 ± 1.2 b	5.5 ± 0.8 a
tCr	3.2 ± 0.4 ab	3.3 ± 0.5 a	2.9 ± 0.3 b	3.4 ± 0.6 a
tNAA	3.3 ± 0.5 ab	3.1 ± 0.3 ab	3.0 ± 0.3 a	3.3 ± 0.5 b

Note. Values are means ± SD. Compact letter displays indicate Tukey-adjusted pairwise differences in CRLB values. Sessions sharing at least one letter do not differ significantly, whereas sessions with no shared letters differ significantly ($p < .05$). Abbreviations: D1M1 = day 1, measurement 1; D1M2 = day 1, measurement 2; D2M1 = day 2, measurement 1; D2M2 = day 2, measurement 2; CRLB = Cramér-Rao lower bound.

Table S7. *CRLB omnibus ANOVA results*

Metabolite	numDF	denDF	F-value	p-value
GSH	3	49	7.71	<.001
Glc_Tau	3	49	4.65	0.006
Gln	3	49	6.13	0.001
Glu	3	49	9.62	<.001
Glx	3	49	7.90	<.001
mI	3	49	2.98	0.040
tCho	3	49	3.43	0.024

Metabolite	numDF	denDF	F-value	p-value
tCr	3	49	4.58	0.007
tNAA	3	49	3.27	0.029

Note. Omnibus tests are from linear mixed-effects models with Measurement as fixed effect and participant-specific random intercepts. CRLB = Cramér-Rao lower bound.

***Table S8**. CRLB pairwise comparisons*

Metabolite	Contrast	Estimate	SE	df	t-stat	p-value
GSH	D1M1 vs D1M2	-0.25	1.15	49	-0.21	0.996
GSH	D1M1 vs D2M1	2.63	1.12	49	2.35	0.100
GSH	D1M1 vs D2M2	-2.65	1.10	49	-2.41	0.088
GSH	D1M2 vs D2M1	2.87	1.16	49	2.49	0.075
GSH	D1M2 vs D2M2	-2.40	1.14	49	-2.11	0.165
GSH	D2M1 vs D2M2	-5.27	1.10	49	-4.80	<.001
Glc_Tau	D1M1 vs D1M2	-0.69	0.78	49	-0.88	0.814
Glc_Tau	D1M1 vs D2M1	1.39	0.76	49	1.83	0.272
Glc_Tau	D1M1 vs D2M2	-1.28	0.75	49	-1.72	0.324
Glc_Tau	D1M2 vs D2M1	2.08	0.79	49	2.64	0.052
Glc_Tau	D1M2 vs D2M2	-0.59	0.77	49	-0.77	0.869
Glc_Tau	D2M1 vs D2M2	-2.67	0.75	49	-3.58	0.004
Gln	D1M1 vs D1M2	0.73	1.25	49	0.58	0.937
Gln	D1M1 vs D2M1	1.88	1.21	49	1.54	0.420
Gln	D1M1 vs D2M2	-3.03	1.20	49	-2.53	0.068
Gln	D1M2 vs D2M1	1.15	1.25	49	0.92	0.796
Gln	D1M2 vs D2M2	-3.75	1.24	49	-3.03	0.019
Gln	D2M1 vs D2M2	-4.90	1.20	49	-4.09	<.001
Glu	D1M1 vs D1M2	-0.20	0.25	49	-0.79	0.859
Glu	D1M1 vs D2M1	0.62	0.24	49	2.53	0.067
Glu	D1M1 vs D2M2	-0.65	0.24	49	-2.72	0.043
Glu	D1M2 vs D2M1	0.82	0.25	49	3.22	0.012
Glu	D1M2 vs D2M2	-0.45	0.25	49	-1.82	0.274
Glu	D2M1 vs D2M2	-1.27	0.24	49	-5.31	<.001

Metabolite	Contrast	Estimate	SE	df	t-stat	p-value
Glx	D1M1 vs D1M2	-0.04	0.23	49	-0.16	0.998
Glx	D1M1 vs D2M1	0.37	0.23	49	1.62	0.378
Glx	D1M1 vs D2M2	-0.69	0.22	49	-3.11	0.016
Glx	D1M2 vs D2M1	0.41	0.24	49	1.72	0.323
Glx	D1M2 vs D2M2	-0.66	0.23	49	-2.83	0.033
Glx	D2M1 vs D2M2	-1.06	0.22	49	-4.76	<.001
mI	D1M1 vs D1M2	0.67	0.65	49	1.03	0.735
mI	D1M1 vs D2M1	1.58	0.63	49	2.50	0.072
mI	D1M1 vs D2M2	-0.06	0.62	49	-0.10	1.000
mI	D1M2 vs D2M1	0.91	0.66	49	1.39	0.510
mI	D1M2 vs D2M2	-0.73	0.64	49	-1.13	0.672
mI	D2M1 vs D2M2	-1.64	0.62	49	-2.65	0.051
tCho	D1M1 vs D1M2	-0.11	0.26	49	-0.43	0.974
tCho	D1M1 vs D2M1	0.60	0.26	49	2.33	0.105
tCho	D1M1 vs D2M2	-0.10	0.25	49	-0.39	0.980
tCho	D1M2 vs D2M1	0.71	0.27	49	2.67	0.049
tCho	D1M2 vs D2M2	0.01	0.26	49	0.06	1.000
tCho	D2M1 vs D2M2	-0.70	0.25	49	-2.76	0.039
tCr	D1M1 vs D1M2	-0.15	0.16	49	-0.94	0.784
tCr	D1M1 vs D2M1	0.27	0.15	49	1.80	0.285
tCr	D1M1 vs D2M2	-0.25	0.15	49	-1.70	0.337
tCr	D1M2 vs D2M1	0.42	0.16	49	2.68	0.047
tCr	D1M2 vs D2M2	-0.11	0.16	49	-0.69	0.899
tCr	D2M1 vs D2M2	-0.53	0.15	49	-3.52	0.005
tNAA	D1M1 vs D1M2	0.15	0.11	49	1.38	0.516
tNAA	D1M1 vs D2M1	0.28	0.11	49	2.57	0.062
tNAA	D1M1 vs D2M2	-0.01	0.11	49	-0.10	1.000
tNAA	D1M2 vs D2M1	0.12	0.11	49	1.10	0.692
tNAA	D1M2 vs D2M2	-0.16	0.11	49	-1.49	0.451

Metabolite	Contrast	Estimate	SE	df	t-stat	p-value
tNAA	D2M1 vs D2M2	-0.29	0.11	49	-2.71	0.044

Note. Pairwise comparisons are Tukey-adjusted estimated marginal mean contrasts from the linear mixed-effects models. CRLB = Cramér-Rao lower bound; SE = standard error.

2.5.1 Metabolite concentrations

Table S9. *Metabolite concentrations*

	Water-referenced				Creatine-referenced			
**Metabolite**	**D1M1**	**D1M2**	**D2M1**	**D2M2**	**D1M1**	**D1M2**	**D2M1**	**D2M2**
GSH	3.18±0.55	3.14±0.54	3.12±0.31	2.84±0.62	0.29±0.06	0.28±0.06	0.29±0.05	0.26±0.06
Glc_Tau	4.53±0.70	4.42±0.95	4.31±0.82	4.16±0.68	0.44±0.08	0.43±0.10	0.42±0.08	0.41±0.07
Gln	4.36±0.70	4.53±1.02	4.04±0.71	3.83±0.72	0.41±0.07	0.43±0.09	0.39±0.07	0.37±0.06
Glu	13.61±1.23	13.27±1.27	12.80±1.07	12.73±1.32	1.26±0.10	1.22±0.09	1.21±0.09	1.20±0.09
Glx	17.87±1.25	17.71±1.77	16.74±1.46	16.46±1.70	1.67±0.10	1.65±0.13	1.60±0.12	1.56±0.11
mI	9.68±1.59	10.06±1.27	9.75±1.29	9.70±1.03	0.98±0.15	1.02±0.14	1.01±0.12	1.00±0.11
tCho	2.93±0.30	2.91±0.34	3.02±0.30	2.97±0.28	0.30±0.04	0.30±0.04	0.32±0.03	0.31±0.03
tCr	10.43±0.69	10.46±0.71	10.23±0.77	10.26±0.79				
tNAA	12.59±0.66	12.88±0.66	12.48±0.76	12.63±0.61	1.29±0.07	1.31±0.06	1.30±0.11	1.31±0.11

Note. Values are means ± SD across participants.

Table S10. *Metabolite concentration omnibus results*

Metabolite	Reference	numDF	denDF	F-value	p-value
GSH	Creatine-referenced	3	49	1.39	0.257
GSH	Water-referenced	3	49	1.81	0.157
Glc_Tau	Creatine-referenced	3	49	0.91	0.444
Glc_Tau	Water-referenced	3	49	1.23	0.309
Gln	Creatine-referenced	3	49	2.78	0.051
Gln	Water-referenced	3	49	3.23	0.030
Glu	Creatine-referenced	3	49	2.52	0.068
Glu	Water-referenced	3	49	4.02	0.012
Glx	Creatine-referenced	3	49	5.52	0.002
Glx	Water-referenced	3	49	6.49	<.001
mI	Creatine-referenced	3	49	0.46	0.710
mI	Water-referenced	3	49	0.32	0.811

Metabolite	Reference	numDF	denDF	F-value	p-value
tCho	Creatine-referenced	3	49	3.10	0.035
tCho	Water-referenced	3	49	1.44	0.243
tCr	Water-referenced	3	49	0.97	0.414
tNAA	Creatine-referenced	3	49	1.37	0.263
tNAA	Water-referenced	3	49	1.98	0.130

Note. Omnibus tests are from linear mixed-effects models with Measurement as fixed effect and participant-specific random intercepts. Models were fitted separately for each metabolite and reference scheme.

Table S11. *Metabolite concentration pairwise comparisons*

Metabolite	Reference	Contrast	Estimate	p-value
GSH	Creatine-referenced	D1M1 vs D1M2	0.00	1.000
GSH	Creatine-referenced	D1M1 vs D2M1	-0.00	1.000
GSH	Creatine-referenced	D1M1 vs D2M2	0.03	0.350
GSH	Creatine-referenced	D1M2 vs D2M1	-0.00	0.998
GSH	Creatine-referenced	D1M2 vs D2M2	0.02	0.442
GSH	Creatine-referenced	D2M1 vs D2M2	0.03	0.317
GSH	Water-referenced	D1M1 vs D1M2	0.03	0.998
GSH	Water-referenced	D1M1 vs D2M1	0.06	0.979
GSH	Water-referenced	D1M1 vs D2M2	0.33	0.176
GSH	Water-referenced	D1M2 vs D2M1	0.03	0.997
GSH	Water-referenced	D1M2 vs D2M2	0.30	0.278
GSH	Water-referenced	D2M1 vs D2M2	0.27	0.348
Glc_Tau	Creatine-referenced	D1M1 vs D1M2	0.01	0.941
Glc_Tau	Creatine-referenced	D1M1 vs D2M1	0.01	0.931
Glc_Tau	Creatine-referenced	D1M1 vs D2M2	0.03	0.377
Glc_Tau	Creatine-referenced	D1M2 vs D2M1	0.00	1.000
Glc_Tau	Creatine-referenced	D1M2 vs D2M2	0.02	0.758
Glc_Tau	Creatine-referenced	D2M1 vs D2M2	0.02	0.745
Glc_Tau	Water-referenced	D1M1 vs D1M2	0.14	0.918
Glc_Tau	Water-referenced	D1M1 vs D2M1	0.22	0.722
Glc_Tau	Water-referenced	D1M1 vs D2M2	0.39	0.249
Glc_Tau	Water-referenced	D1M2 vs D2M1	0.08	0.982
Glc_Tau	Water-referenced	D1M2 vs D2M2	0.25	0.651
Glc_Tau	Water-referenced	D2M1 vs D2M2	0.17	0.846
Gln	Creatine-referenced	D1M1 vs D1M2	-0.01	0.927
Gln	Creatine-referenced	D1M1 vs D2M1	0.02	0.717
Gln	Creatine-referenced	D1M1 vs D2M2	0.05	0.166
Gln	Creatine-referenced	D1M2 vs D2M1	0.04	0.368
Gln	Creatine-referenced	D1M2 vs D2M2	0.06	0.050
Gln	Creatine-referenced	D2M1 vs D2M2	0.02	0.732
Gln	Water-referenced	D1M1 vs D1M2	-0.16	0.925
Gln	Water-referenced	D1M1 vs D2M1	0.33	0.540
Gln	Water-referenced	D1M1 vs D2M2	0.54	0.128

Metabolite	Reference	Contrast	Estimate	p-value
Gln	Water-referenced	D1M2 vs D2M1	0.49	0.233
Gln	Water-referenced	D1M2 vs D2M2	0.70	0.036
Gln	Water-referenced	D2M1 vs D2M2	0.21	0.819
Glu	Creatine-referenced	D1M1 vs D1M2	0.03	0.633
Glu	Creatine-referenced	D1M1 vs D2M1	0.05	0.184
Glu	Creatine-referenced	D1M1 vs D2M2	0.06	0.058
Glu	Creatine-referenced	D1M2 vs D2M1	0.02	0.859
Glu	Creatine-referenced	D1M2 vs D2M2	0.03	0.571
Glu	Creatine-referenced	D2M1 vs D2M2	0.01	0.956
Glu	Water-referenced	D1M1 vs D1M2	0.33	0.710
Glu	Water-referenced	D1M1 vs D2M1	0.85	0.031
Glu	Water-referenced	D1M1 vs D2M2	0.86	0.024
Glu	Water-referenced	D1M2 vs D2M1	0.52	0.342
Glu	Water-referenced	D1M2 vs D2M2	0.53	0.303
Glu	Water-referenced	D2M1 vs D2M2	0.01	1.000
Glx	Creatine-referenced	D1M1 vs D1M2	0.02	0.957
Glx	Creatine-referenced	D1M1 vs D2M1	0.08	0.089
Glx	Creatine-referenced	D1M1 vs D2M2	0.11	0.004
Glx	Creatine-referenced	D1M2 vs D2M1	0.06	0.279
Glx	Creatine-referenced	D1M2 vs D2M2	0.10	0.022
Glx	Creatine-referenced	D2M1 vs D2M2	0.04	0.640
Glx	Water-referenced	D1M1 vs D1M2	0.17	0.974
Glx	Water-referenced	D1M1 vs D2M1	1.18	0.021
Glx	Water-referenced	D1M1 vs D2M2	1.42	0.003
Glx	Water-referenced	D1M2 vs D2M1	1.01	0.076
Glx	Water-referenced	D1M2 vs D2M2	1.24	0.016
Glx	Water-referenced	D2M1 vs D2M2	0.24	0.928
mI	Creatine-referenced	D1M1 vs D1M2	-0.03	0.765
mI	Creatine-referenced	D1M1 vs D2M1	-0.03	0.727
mI	Creatine-referenced	D1M1 vs D2M2	-0.02	0.932
mI	Creatine-referenced	D1M2 vs D2M1	-0.00	1.000
mI	Creatine-referenced	D1M2 vs D2M2	0.01	0.977
mI	Creatine-referenced	D2M1 vs D2M2	0.01	0.967
mI	Water-referenced	D1M1 vs D1M2	-0.23	0.850
mI	Water-referenced	D1M1 vs D2M1	-0.04	0.999
mI	Water-referenced	D1M1 vs D2M2	0.02	1.000
mI	Water-referenced	D1M2 vs D2M1	0.19	0.912
mI	Water-referenced	D1M2 vs D2M2	0.25	0.806
mI	Water-referenced	D2M1 vs D2M2	0.06	0.995
tCho	Creatine-referenced	D1M1 vs D1M2	0.00	1.000
tCho	Creatine-referenced	D1M1 vs D2M1	-0.02	0.060
tCho	Creatine-referenced	D1M1 vs D2M2	-0.01	0.421
tCho	Creatine-referenced	D1M2 vs D2M1	-0.02	0.069
tCho	Creatine-referenced	D1M2 vs D2M2	-0.01	0.433

Metabolite	Reference	Contrast	Estimate	p-value
tCho	Creatine-referenced	D2M1 vs D2M2	0.01	0.699
tCho	Water-referenced	D1M1 vs D1M2	0.02	0.994
tCho	Water-referenced	D1M1 vs D2M1	-0.10	0.369
tCho	Water-referenced	D1M1 vs D2M2	-0.06	0.781
tCho	Water-referenced	D1M2 vs D2M1	-0.12	0.276
tCho	Water-referenced	D1M2 vs D2M2	-0.07	0.654
tCho	Water-referenced	D2M1 vs D2M2	0.04	0.889
tCr	Water-referenced	D1M1 vs D1M2	0.02	1.000
tCr	Water-referenced	D1M1 vs D2M1	0.23	0.455
tCr	Water-referenced	D1M1 vs D2M2	0.15	0.762
tCr	Water-referenced	D1M2 vs D2M1	0.22	0.556
tCr	Water-referenced	D1M2 vs D2M2	0.13	0.839
tCr	Water-referenced	D2M1 vs D2M2	-0.08	0.951
tNAA	Creatine-referenced	D1M1 vs D1M2	-0.04	0.302
tNAA	Creatine-referenced	D1M1 vs D2M1	-0.03	0.562
tNAA	Creatine-referenced	D1M1 vs D2M2	-0.03	0.330
tNAA	Creatine-referenced	D1M2 vs D2M1	0.01	0.961
tNAA	Creatine-referenced	D1M2 vs D2M2	0.00	0.999
tNAA	Creatine-referenced	D2M1 vs D2M2	-0.01	0.981
tNAA	Water-referenced	D1M1 vs D1M2	-0.33	0.228
tNAA	Water-referenced	D1M1 vs D2M1	0.06	0.983
tNAA	Water-referenced	D1M1 vs D2M2	-0.11	0.916
tNAA	Water-referenced	D1M2 vs D2M1	0.39	0.119
tNAA	Water-referenced	D1M2 vs D2M2	0.23	0.551
tNAA	Water-referenced	D2M1 vs D2M2	-0.17	0.737

Note. Pairwise comparisons are Tukey-adjusted estimated marginal mean contrasts from the linear mixed-effects models. Estimates represent pairwise differences between sessions.

***Table S12**. Overview of session effects on metabolite concentrations*

Metabolite	Reference	F-value	p-value	Significant contrasts
GSH	Water	1.81	0.157	—
Glc_Tau	Water	1.23	0.309	—
Gln	Water	3.23	0.030	D1M2 > D2M2
Glu	Water	4.02	0.012	D1M1 > D2M1; D1M1 > D2M2
Glx	Water	6.49	<.001	D1M1 > D2M1; D1M1 > D2M2; D1M2 > D2M2
mI	Water	0.32	0.811	—
tCho	Water	1.44	0.243	—
tCr	Water	0.97	0.414	—
tNAA	Water	1.98	0.130	—
GSH	tCr	1.39	0.257	—
Glc_Tau	tCr	0.91	0.444	—
Gln	tCr	2.78	0.051	—
Glu	tCr	2.52	0.068	—
Glx	tCr	5.52	0.002	D1M1 > D2M2; D1M2 > D2M2

Metabolite	Reference	F-value	p-value	Significant contrasts
mI	tCr	0.46	0.710	—
tCho	tCr	3.10	0.035	—
tNAA	tCr	1.37	0.263	—

Note. Omnibus tests are from linear mixed-effects models with Measurement as fixed effect and participant-specific random intercepts. Significant contrasts are Tukey-adjusted pairwise comparisons between sessions. Direction signs indicate the direction of the estimated pairwise difference. tCr = creatine-referenced; Water = water-referenced.

2.6 Exploratory Voxel-Overlap Analyses

***Table S13**. Voxel placement consistency*

	Mean	SD	Minimum	Maximum
All sessions	0.793	0.073	0.526	0.932
Same day	0.824	0.050	0.718	0.922
Different days	0.780	0.064	0.615	0.871

Note. Dice coefficients across participants. "All sessions" includes all six pairwise combinations between the four acquisitions (1–2, 1–3, 1–4, 2–3, 2–4, and 3–4). "Same day" refers to participant-level averages across within-day pairs, and "Different days" refers to participant-level averages across between-day pairs. Group-level descriptive statistics were then calculated across participants. SD = standard deviation.

***Table S14**. Association between voxel overlap and metabolite concentration differences*

Metabolite	Dice β	SE	95% CI	t-stat	p-value
Gln_tcr	-0.005	0.090	[-0.186, 0.175]	-0.060	0.953
Glu_tcr	-0.138	0.096	[-0.329, 0.054]	-1.430	0.157
GSH_tcr	0.052	0.064	[-0.075, 0.180]	0.820	0.415
mI_tcr	-0.023	0.110	[-0.241, 0.195]	-0.209	0.835
tCho_tcr	-0.002	0.027	[-0.055, 0.052]	-0.063	0.950
tNAA_tcr	-0.041	0.076	[-0.192, 0.110]	-0.542	0.589
Glx_tcr	-0.021	0.117	[-0.253, 0.211]	-0.179	0.858
Glc_Tau_tcr	0.113	0.081	[-0.048, 0.273]	1.399	0.166
Gln_water	0.609	1.009	[-1.401, 2.618]	0.603	0.548
Glu_water	-0.284	1.162	[-2.597, 2.029]	-0.244	0.808
GSH_water	0.073	0.648	[-1.218, 1.363]	0.112	0.911

Metabolite	Dice β	SE	95% CI	t-stat	p-value
mI_water	0.571	1.028	[-1.476, 2.617]	0.555	0.580
tCho_water	0.012	0.238	[-0.462, 0.485]	0.049	0.961
tCr_water	-0.434	0.629	[-1.687, 0.818]	-0.691	0.492
**tNAA_water**	**-1.733**	**0.656**	**[-3.040, -0.426]**	**-2.641**	**0.010**
Glx_water	0.504	1.548	[-2.579, 3.587]	0.325	0.746
Glc_Tau_water	0.445	0.870	[-1.287, 2.176]	0.511	0.611

Note. Results from exploratory linear mixed-effects models examining the association between voxel overlap, indexed by the Dice coefficient, and absolute between-measurement differences in metabolite concentrations. Each row represents a separate model for one metabolite and quantification scheme. Negative β values indicate that higher voxel overlap was associated with smaller between-measurement differences. SE = standard error. CI = confidence interval

2.6.1 Voxel overlap and water-referenced tNAA differences

***Figure S3**. Association between voxel overlap and water-referenced tNAA differences.*

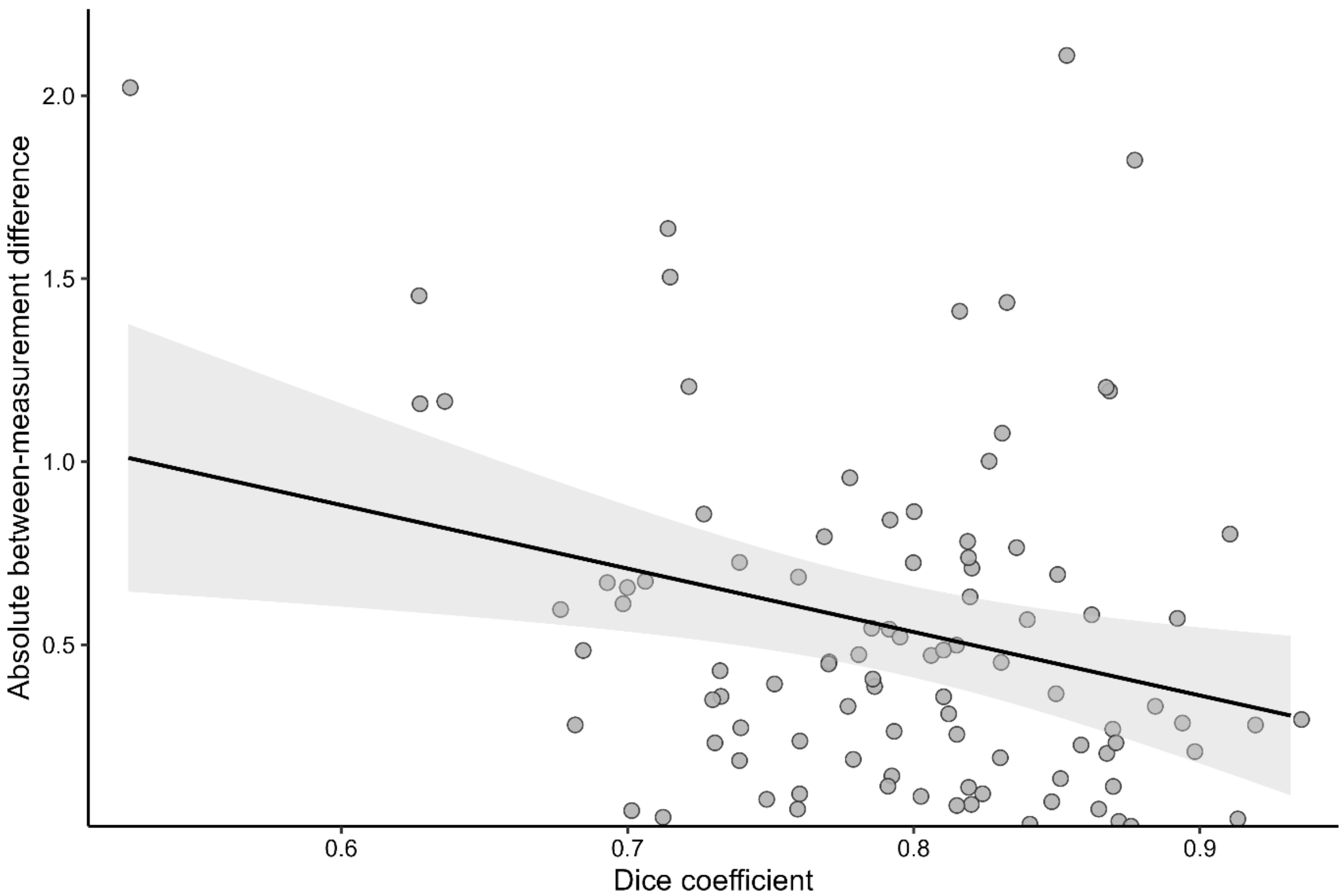

Note. Association between voxel overlap and water-referenced tNAA differences. Points represent pairwise observations, with Dice coefficients indexing voxel overlap and absolute differences indexing the corresponding between-measurement difference in water-referenced tNAA concentration. The fitted

line and 95% confidence band represent fixed-effect predictions from the linear mixed-effects model with participant-specific random intercepts.

Table S15. *Association between voxel overlap and metabolite concentration differences after excluding two participants with low voxel overlap*

Metabolite	Dice β	SE	95% CI	t-stat	p-value
Gln_tcr	-0.040	0.113	[-0.265, 0.186]	-0.349	0.728
Glu_tcr	-0.233	0.119	[-0.469, 0.004]	-1.964	0.054
GSH_tcr	0.096	0.071	[-0.045, 0.238]	1.354	0.180
mI_tcr	0.076	0.133	[-0.189, 0.341]	0.574	0.568
tCho_tcr	0.012	0.032	[-0.052, 0.077]	0.385	0.701
tNAA_tcr	-0.030	0.090	[-0.209, 0.150]	-0.328	0.744
Glx_tcr	-0.028	0.145	[-0.318, 0.261]	-0.196	0.845
Glc_Tau_tcr	0.159	0.099	[-0.039, 0.357]	1.598	0.115
Gln_water	-0.005	1.274	[-2.546, 2.536]	-0.004	0.997
Glu_water	-0.132	1.424	[-2.972, 2.707]	-0.093	0.926
GSH_water	0.578	0.761	[-0.940, 2.095]	0.759	0.450
mI_water	0.670	1.264	[-1.851, 3.191]	0.530	0.598
tCho_water	-0.124	0.300	[-0.723, 0.474]	-0.415	0.680
tCr_water	0.049	0.738	[-1.423, 1.522]	0.067	0.947
tNAA_water	-0.551	0.775	[-2.097, 0.995]	-0.711	0.480
Glx_water	0.429	1.931	[-3.423, 4.280]	0.222	0.825
Glc_Tau_water	1.243	1.092	[-0.936, 3.421]	1.138	0.259

Note. Results from exploratory linear mixed-effects models examining the association between voxel overlap, indexed by the Dice coefficient, and absolute between-measurement differences in metabolite concentrations after excluding participants 3 and 11. Each row represents a separate model for one metabolite and quantification scheme. Negative β values indicate that higher voxel overlap was associated with smaller between-measurement differences.

Figure S4*. Association between voxel overlap and water-referenced tNAA differences after excluding two participants with low voxel overlap*

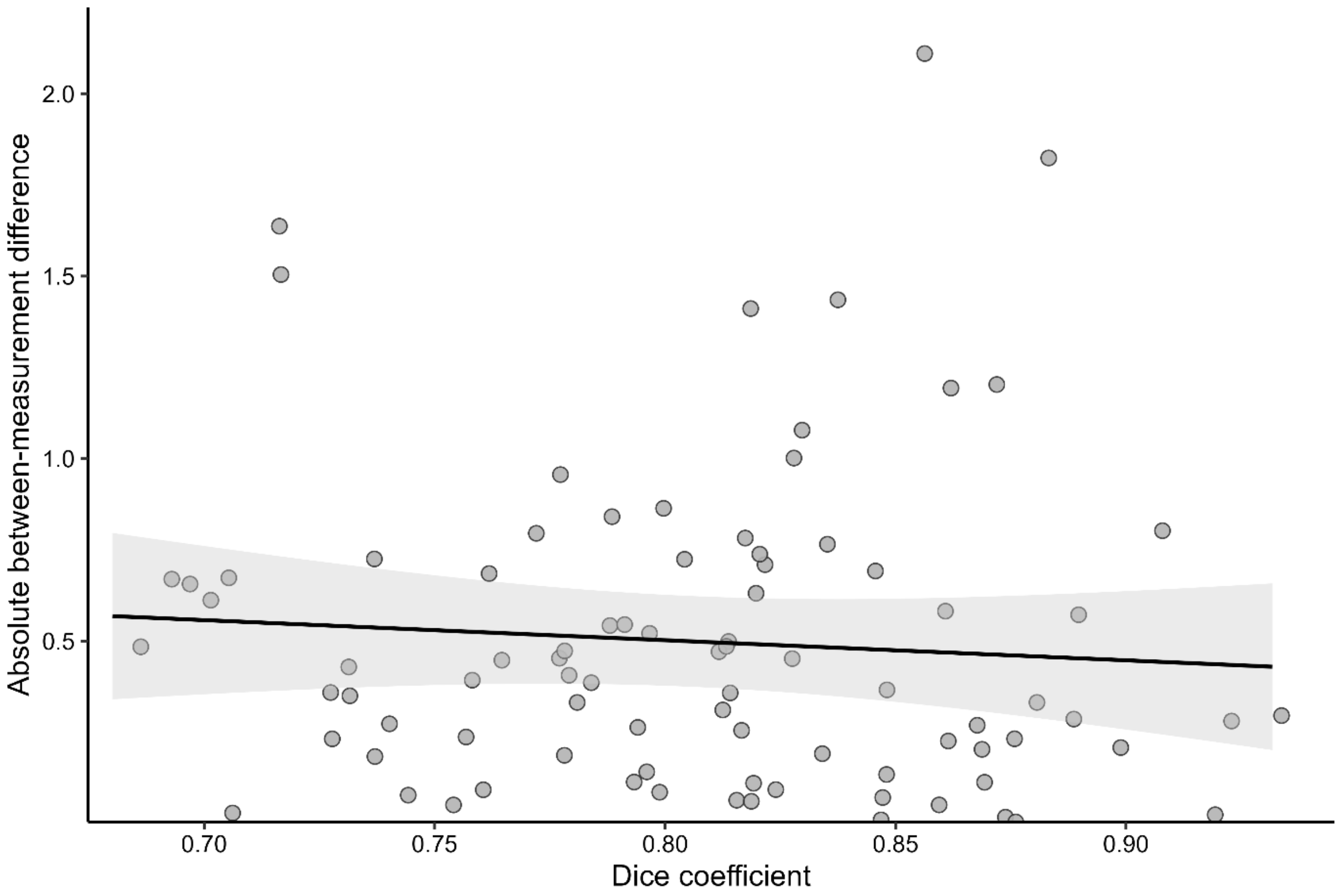

Note. Association between voxel overlap and water-referenced tNAA differences after excluding participants 3 and 11. Points represent pairwise observations, with Dice coefficients indexing voxel overlap between two measurements and absolute differences indexing the corresponding between-measurement difference in water-referenced tNAA concentration. The fitted line and 95% confidence band represent the fixed-effect prediction from the linear mixed-effects model with participant-specific random intercepts.

Supplementary references

Ashburner, J., & Friston, K. J. (2005). Unified segmentation. *NeuroImage*, *26*(3), 839–851. https://doi.org/10.1016/j.neuroimage.2005.02.018

Bai, X., Harris, A. D., Gong, T., Puts, N. A. J., Wang, G., Schär, M., Barker, P. B., & Edden, R. A. E. (2017). Voxel Placement Precision for GABA-Edited Magnetic Resonance Spectroscopy. *Open Journal of Radiology*, *7*(1), 35–44. https://doi.org/10.4236/ojrad.2017.71004

Crum, W. R., Camara, O., & Hill, D. L. G. (2006). Generalized Overlap Measures for Evaluation and Validation in Medical Image Analysis. *IEEE Transactions on Medical Imaging*, *25*(11), 1451–1461. https://doi.org/10.1109/TMI.2006.880587

Zijdenbos, A. P., Dawant, B. M., Margolin, R. A., & Palmer, A. C. (1994). Morphometric analysis of white matter lesions in MR images: Method and validation. *IEEE Transactions on Medical Imaging*, *13*(4), 716–724. https://doi.org/10.1109/42.363096

Zou, K. H., Warfield, S. K., Bharatha, A., Tempany, C. M. C., Kaus, M. R., Haker, S. J., Wells, W. M., Jolesz, F. A., & Kikinis, R. (2004). Statistical validation of image segmentation quality based on a spatial overlap index1. *Academic Radiology*, *11*(2), 178–189. https://doi.org/10.1016/S1076-6332(03)00671-8
